# Investigation of DNA damage response and apoptotic gene methylation pattern in sporadic breast tumors using high throughput quantitative DNA methylation analysis technology

**DOI:** 10.1186/1476-4598-9-303

**Published:** 2010-11-23

**Authors:** Ranjana Pal, Niloo Srivastava, Rupali Chopra, Sailesh Gochhait, Pawan Gupta, Neeraj Prakash, Gaurav Agarwal, Rameshwar NK Bamezai

**Affiliations:** 1 National Centre of Applied Human Genetics, School of Life Sciences, Jawaharlal Nehru University, Aruna Asafali Road, New Delhi-110067, India; 2 Dharamshila Cancer Hospital and Research Centre, Dharamshila Marg, Vasundhara Enclave, Delhi-96; 3 Rajiv Gandhi Cancer Institute and Research Center, Sector V, Rohini, New Delhi - 110085, India; 4 Sanjay Gandhi Postgraduate Institute of Medical Sciences, Raebareli Road, Lucknow-226 014, India

## Abstract

**Background-:**

Sporadic breast cancer like many other cancers is proposed to be a manifestation of abnormal genetic and epigenetic changes. For the past decade our laboratory has identified genes involved in DNA damage response (DDR), apoptosis and immunesurvelliance pathways to influence sporadic breast cancer risk in north Indian population. Further to enhance our knowledge at the epigenetic level, we performed DNA methylation study involving 17 gene promoter regions belonging to DNA damage response (DDR) and death receptor apoptotic pathway in 162 paired normal and cancerous breast tissues from 81 sporadic breast cancer patients, using a high throughput quantitative DNA methylation analysis technology.

**Results-:**

The study identified five genes with statistically significant difference between normal and tumor tissues. Hypermethylation of *DR5 *(P = 0.001)*, DCR1 *(P = 0.00001)*, DCR2 *(P = 0.0000000005) and *BRCA2 *(P = 0.007) and hypomethylation of *DR4 *(P = 0.011) in sporadic breast tumor tissues suggested a weak/aberrant activation of the DDR/apoptotic pathway in breast tumorigenesis. Negative correlation was observed between methylation status and transcript expression levels for *TRAIL*, *DR4*, *CASP8*, *ATM*, *CHEK2*, *BRCA1 *and *BRCA2 *CpG sites. Categorization of the gene methylation with respect to the clinicopathological parameters showed an increase in aberrant methylation pattern in advanced tumors. These uncharacteristic methylation patterns corresponded with decreased death receptor apoptosis (P = 0.047) and DNA damage repair potential (P = 0.004) in advanced tumors. The observation of BRCA2 -26 G/A 5'UTR polymorphism concomitant with the presence of methylation in the promoter region was novel and emerged as a strong candidate for susceptibility to sporadic breast tumors.

**Conclusion-:**

Our study indicates that methylation of DDR-apoptotic gene promoters in sporadic breast cancer is not a random phenomenon. Progressive epigenetic alterations in advancing tumors result in aberrant DDR-apoptotic pathway thereby promoting tumor development. We propose, since pathological epigenetic changes of the DDR-apoptotic genes are reversible modifications, these could further be targeted for therapeutic interventions.

## Introduction

Breast cancer is the leading cause of cancer mortality among women aged between 20 - 59 years; second leading cause of cancer mortality among all women [1]. The sporadic form represents almost 90% of the total number of breast cancer cases, genetic etiology of which is least understood and the molecular mechanism underlying the onset and progression not clear. It is, however, believed to be a manifestation of abnormal genetic as well as epigenetic changes [2-4] along with the influence of dietary, environmental and physical factors [5]. Previous studies from our laboratory have identified genes involved in DNA damage response (DDR), apoptosis and immunesurvelliance pathways such as polymorphisms in BRCA2, p53 [6], IFNG [7], TGFB1 [8], TRAIL [9] and mDNA [10], somatic mutations in IL6 [11] and mDNA [12], copy number variation in H2AX [13] and aberrant expression of DDR pathway [14] to influence sporadic breast cancer risk in north Indian population. Since, studies have identified *de-novo *methyltransferases, DNMT3b, over-expressed in breast tumors [15], indicating an involvement of epigenetic modifications in oncogenesis, breast cancer susceptibility genes, identified under the categories of DNA damage response (DDR) and apoptosis related genes could be a major target of epigenetic inactivation in sporadic breast cancers. Also, numerous studies have shown that large scale methylation profiling of multiple CpG sites could prove essential to provide comprehensive information on DNA methylation changes occurring during neoplastic transformation [16-24].

**Figure 1:**
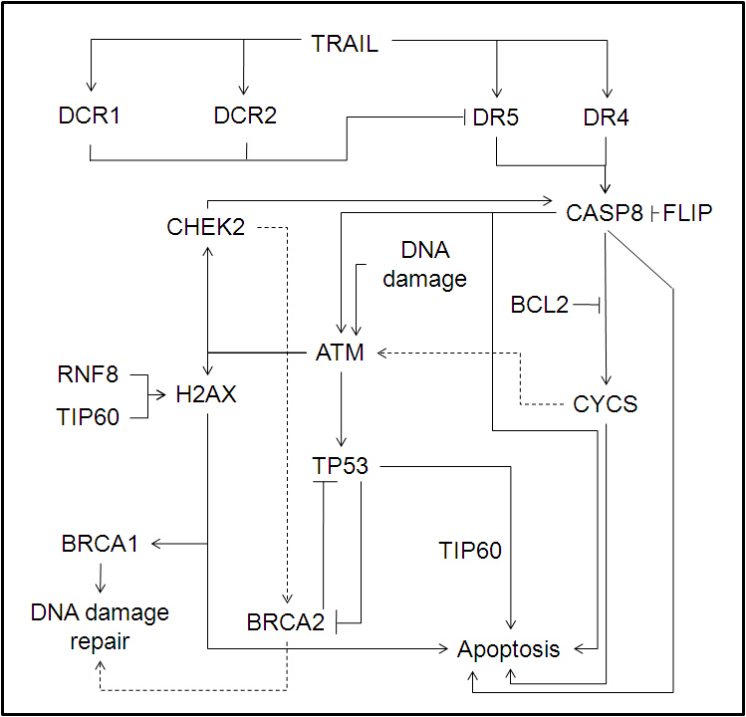
**Crosstalk between DNA damage response and Death Receptor apoptotic pathways**.

In cancer cells, apoptosis induced by extrinsic pathway complements the intrinsic pathway [25]. The extrinsic signal transduction pathway is activated by TRAIL that binds two types of receptors: DR4/5 and DCR1/2 [25]. Binding of TRAIL to DR4 and/or DR5 results in receptor oligomerization and subsequent activation of CASP8 resulting in apoptosis [26]. Similarly, CASP8 can also activate the intrinsic apoptotic pathway causing the release of CYCS from the mitochondria which serves to amplify the death receptor apoptotic signal [26]. On the contrary, decoy receptors, FLIP and BCL2 block the apoptotic signal transduction and promote survival [25,27]. Recent studies have also identified the involvement of death receptors in activating the DNA damage response (DDR) pathway. The activation of ATM, CHEK2 and H2AX in response to TRAIL acts as a positive feedback loop involving the activation of caspases [28,29]. Additionally, RNF8 ubiquitinates H2AX thus, enhancing its activation [30]. H2AX, ATM and/or CHEK2 result in phosphorylation and activation of P53 and regulating DNA repair as well as apoptosis [31]. Although P53 is not essential for TRAIL mediated apoptosis [32,33], it can cause transcriptional induction of pro-apoptotic genes such as *TRAIL, DR4, DR5 *[34-36] and antagonize pro-survival genes, *FLIP *and *BCL2 *[37,38]. Further, acetylation of P53 by TIP60, also known as KAT5 promotes apoptosis by preferentially transactivating pro-apoptotic genes [39]. Moreover, DNA damage response also results in activation of BRCA1 and BRCA2 that are involved in the repair of damaged DNA [40] (Figure 1).

Although, several methylation studies have been carried out for *DR4, DR5, DCR1, DCR2, CASP8, TP53, BRCA1 *and *BRCA2 *in various types of cancer, none of them provide a holistic view of the DDR - apoptotic pathway [21,41-45]. Therefore, after investigating the breast tumor at the genetic and expression level, we for the first time provide an integrated information on methylation patterns of the DDR and death receptor apoptotic pathway genes (*TRAIL, DR4, DR5, DCR1, DCR2, CASP8, FLIP, BCL2, CYCS, ATM, TP53, BRCA1, BRCA2, H2AX, RNF8, TIP60 *and *CHEK2*) in sporadic breast tumors, using high throughput automated MALDI TOF [17-19,22]. The study further dissects the role of methylation of candidate genes in sporadic breast tumorigenesis, its interaction with functional BRCA2 -26 SNP and the status of pro- and anti- apoptotic as well as DNA damage repair gene expression in breast tumor tissues.

## Materials and methods

### Samples

A total of 162 tissue samples (81 ductal carcinoma tissues and 81 adjacent normal tissues, both of parenchymal origin) were collected from 81 patients with sporadic breast cancer from Dharamshila Cancer Hospital and Research Centre, New Delhi; Rajiv Gandhi Cancer Institute and Research Center, New Delhi and Sanjay Gandhi Postgraduate Institute of Medical Sciences, Lucknow. The study samples adhered to the REMARK guidelines [46]. The female patients ranged in the age group of 25 to 77 years, with a median of 48 years. None of the studied cases had a hereditary form of breast cancer. Prior approval was obtained from Jawaharlal Nehru University ethical committee and the informed consent taken of the concerned subjects for sample collection and study.

Differentiation between cancerous and normal tissue was based on magnetic resonance imaging, intra-operative gross surgical pathology and tissue histology. Breast tumor patients underwent imaging (mammography, ultra-sonography and MRI) to know the location, size and extent of tumor. Tumor was removed wide of its margin i.e. 1-2 cm envelope of normal breast tissue was left all around the tumor to ensure removal of any microscopic extensions of the tumor into normal breast parenchyma. What we considered as normal tissue was taken 2 cm away from the palpable tumor margin. This fact was further confirmed by frozen section histology and paraffin section histology before labeling the tumor and normal tissues for molecular biology experiments, thereby ensuring the absence of cross contamination.

Patients were classified on the basis of tumor size, nodal status, tumor stage, estrogen and progesterone receptor (ER and PR) status. At the time of diagnosis, 7 patients had stage I disease, 44 patients belonged to stage II, 28 patients to stage III and 2 patients had stage IV disease. ER/PR status and tumor grade could be obtained for 57 out of 81 pairs of tumor tissues studied. Immunohistochemical staining identified 27/24 ER/PR positive patients and 30/33 ER/PR negative patients. Out of 57 patients, 2 patients had grade I tumor, 30 had grade II tumor and 25 had grade III tumor (Additional file 1, Table S1). The samples collected were frozen immediately and stored at -80°C until use. DNA extraction was performed from 0.01 - 0.02 g of tissue sample, using the Genelute Mammalian genomic DNA isolation kit (Sigma Aldrich, St. Louis, Missouri, USA).

### Bisulfite treatment and PCR

The EZ-96 DNA Methylation Kit (Zymo Research, Orange, CA, USA) was used for bisulfite conversion of the target sequences. The C/T conversion reaction was performed using the PCR program as follows: 95°C for 30 sec and 50°C for 15 min, which was repeated for 46 cycles. Primers were designed using epidesigner software (Sequenom, San Diego, CA, USA) to cover the regions with the most CpG sites (Additional file 2, Table S2). Our selected amplicons were mostly located in the promoter region of genes or started from the promoter and ended in the first exon. In PCR amplification, a T7-promoter tag was added to the reverse primer and a 10 mer-tag sequence was added to the forward primer to balance the PCR primer length. The bisulfite treated genomic DNA was amplified using Taq DNA polymerase (Roche Diagnostics, Mannheim, Germany) (4 min at 95°C followed by 45 cycles of 20 sec at 95°C, 30 sec at 62°C, and 1 min at 72°C with a 3 minute final extension). PCR products were analyzed further in Sequenom MassARRAY. Methylated and unmethylated positive control human DNA was procured from Sequenom. Fully methylated DNA was mixed with pure unmethylated DNA in a ratio of 100:0, 60:40, 40:60, and 0:100.

### *In vitro *transcription, T cleavage assay and Mass Spectrometry

Unincorporated dNTPs were dephosphorylated by adding 1.7 μl H_2_O and 0.3U shrimp alkaline phosphatase (Sequenom) followed by incubation at 37°C for 40 min and heat inactivation of shrimp alkaline phosphatase at 85°C for 5 min. In general, 2 μl of the PCR was directly used as a template in a 5 μl transcription reaction. T7 RNA and DNA Polymerase (20U) (Sequenom) was used to incorporate dTTP in the transcripts. Ribonucleotides were used at 1 mmol/l and the dNTP substrate at 2.5 mmol/l. RNaseA enzyme (Sequenom) was added in the same step to cleave the *in vitro *transcripts (T-cleavage assay). The T cleavage reaction was carried out at 37°C for 3 hours and further diluted with H_2_O to a final volume of 27 μl. Conditioning of the phosphate backbone was achieved by adding 6 mg of Clean Resin (Sequenom) before performing MALDI-TOF MS.

22 nl of cleavage reaction was robotically dispensed onto a silicon matrix preloaded chips (SpectroCHIP; Sequenom), and the mass spectra obtained using a MassARRAY. Methylation ratios were generated by the MALDI-TOF and EpiTYPER software v1.0 (Sequenom). The assay was able to discriminate between the methylated and unmethylated components of the positive control according to the ratios.

### Genotype and Expression Analysis

Sequence based analysis of the amplified 5'UTR region of the BRCA2 gene as described earlier [6] for genotype status was performed for 81 samples in which methylation study was carried out. Commercially available Taqman Gene expression Assay system for quantitating transcript level of *TRAIL, DR4, DR5, DCR1, DCR2, CASP8, CASP8L, FLIPL, FLIPS, BCL2, CYCS, ATM, TP53, BRCA1, BRCA2, CHEK2 *[9,14] and *H2AX *(Hs01573336_s1) genes belonging to the DDR and apoptotic pathway were used for studying mRNA expression in 40 representative tumor samples out of the 81 paired normal/tumor samples used in methylation study (Applied Biosystems, Foster City, CA, USA). *GAPDH*, *B-Actin*, *PUM1*, and *MRPL19 *(Applied Biosystems) were used as endogenous controls [9]. Quantitative real-time PCR was carried out using an ABI Prism 7900 Sequence Detection System (Applied Biosystems). Threshold cycle (Ct) numbers were established by using SDS 1.1 RQ software (Applied Biosystems). All the reactions were carried out in duplicates. The normalization factor obtained from GeNorm software was used to compute normalized expression for the target genes [9].

Categorization of the results was done on the basis of pro- versus anti- apoptotic genes involved in death receptor apoptotic pathway [(*TRAIL + DR4 + DR5 + CASP8 + ATM + H2AX + CHEK2 + CYCS)/(DCR1 + DCR2 + CASP8L + FLIPL + FLIPS + BCL2*)] and DNA damage response pathway [*ATM + CHEK2 + BRCA1 + BRCA2 + H2AX*]. Earlier studies from our laboratory have shown that higher level of P53 expression in advanced breast tumors did not reflect in P53 activity in later stages of tumor development [14] and thus was not considered while calculating the DNA damage response signal.

### Statistical Methods

Relative methylation was compared using the Wilcoxon signed-rank test, a nonparametric counterpart of the paired t-test to identify sites with statistically significant difference, between cancerous and paired normal tissues. Using the two-way hierarchical cluster analysis, the CpG fragments for each gene were clustered based on pair-wise Euclidean distances and linkage algorithm for all of the 81 pairs of tissue samples. Clustering of CpG units were plotted along the y axis and samples along the x axis. The procedure was carried out using the heatmap.2 function of the 'gplots' package using the R statistical software. Comparison of methylation between tumor and normal group was performed using Mann-Whitney U test for two groups http://udel.edu/~mcdonald/statkruskalwallis.html. Fisher's exact test and Spearman's correlation coefficient was calculated using the SPSS statistical package, version 13 (SPSS Inc., Chicago, IL, USA). P value was considered significant at and below ≤ 0.05.

## Results

### Semiquantitative methylation profiles of 17 genes in cancerous and normal tissue

In this study, we analyzed the methylation patterns of 17 breast cancer genes in 162 (cancerous and normal) breast tissues from 81 breast tumor patients. For the 17 genes, one amplicon per gene was analyzed and the median amplicon length was 427 bp (range = 265-496 bp). In total we assessed 492 CpGs per sample with a total of 79,704 CpGs in 81 pairs of samples. We removed those CpG sites that could not be detected by MALDI-TOF MS and did not yield successful measurements because of generation of higher and lower mass fragments. CpGs which gave good results for > 90% of the samples were considered; these consisted of 227 CpGs per sample (139 CpG sites or units) equivalent to 36,774 CpGs in 81 pairs of samples equating to 46.14% of the total CpGs (Additional file 3, Table S3).

**Figure 2:**
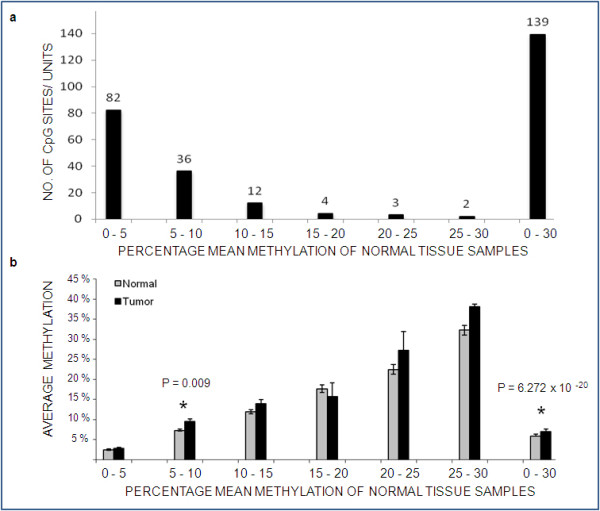
**Binned CpG units and differential methylation.(A)** CpG sites/units binned on the basis of their average methylation value. Each bin contained amplicon within a 5% range of methylation values. Lower bins contained more CpGs in the set of 81 normal breast tissue **(B)** Differential methylation in normal vs. tumor samples across different mean methylation groups based on binning of normal samples. P value was obtained using Mann-Whitney U test for two groups. Note: X - Axis represents groups of mean methylation obtained in normal tissue samples for the study population. For each of the 139 CpG units, the methylation values for the 81 normal tissues were added to obtain mean methylation values. These were then divided into groups of 0% - 5%, 5% - 10%, 10% - 15%, 15% - 20%, 20% - 25% and 25% - 30%.

In normal tissues, 59% of all CpGs showed a mean value of < 5% methylation; 25% showed 5%-10% methylation and the remaining 16% displayed 10%-30% methylation. However, none of the normal tissues studied showed > 30% methylation, suggesting a lower level of methylation in our study group for the candidate genes (Figure 2a). This was in agreement with previous finding for normal tissue samples [18]. Mann-Whitney U test showed average methylation to be significantly higher in tumors whose paired counterpart belonged to the mean methylation group of 5%-10% (P = 0.009). Moreover, average methylation turned out to be highly significant on comparison of whole sporadic breast tumor group with the normal group (P = 6.272 × 10^-20^) (Figure 2b).

Wilcoxon signed rank test followed by Benjamini Hochberg correction for individual CpG sites identified the sites that showed statistically significant difference between normal and tumor samples (P ≤ 0.05) (Table 1). Tumor tissue showed hypomethylation for *DR4, FLIP*, and *RNF8 *and hypermethylation for *DR5, DCR1, DCR2, CASP8, CYCS, BRCA1, BRCA2*, whereas *H2AX*, *TRAIL, BCL2, ATM, CHEK2, TP53 *and *TIP60 *did not show any significant difference between the breast tumor and the corresponding normal tissues. Categorization of the significant CpG units with respect to the transcription start site (TSS) revealed their presence in the promoter, 5'UTR, coding exon 1 and/or intron1-2 regions.

**Table 1:** Wilcoxon signed rank test showing methylation status between breast tumor and adjacent normal tissue

SR. NO.	GENE CpG SITE; N = 81 NOR - TUM PAIRS	P^a^	P^b^	METHYLATION STATUS	LOCATION
**1**	*DR4 *18.19 (-35,-39)	0.010	**0.041**	Hypomethylation	Promoter

**2**	*DR4 *20.21 (-89,-93)	0.007	**0.033**	Hypomethylation	Promoter

**3**	*DR4 *23 (-111)	0.009	**0.041**	Hypomethylation	Promoter

**4**	*DR4 *24 (-137)	0.018	0.069	Hypomethylation	Promoter

**5**	*DR5 *11 (-149)	0.006	**0.031**	Hypermethylation	Promoter

**6**	*DR5 *27 (-363)	0.040	0.149	Hypermethylation	Promoter

**7**	*DR5 *28.29 (-374,-376)	0.0003	**0.003**	Hypermethylation	Promoter

**8**	*DCR1 *4.5.6 (-17,-12,-7)	0.0002	**0.002**	Hypermethylation	Promoter

**9**	*DCR1 *11 (+100)	0.0004	**0.003**	Hypermethylation	5'UTR

**10**	*DCR1 *12 (+110)	0.0003	**0.002**	Hypermethylation	5'UTR

**11**	*DCR1 *13.14.15 (+134,+139,+143)	0.0002	**0.001**	Hypermethylation	5'UTR

**12**	*DCR1 *16 (+154)	0.001	**0.008**	Hypermethylation	5'UTR

**13**	*DCR1 *17 (+166)	0.000008	**0.0001**	Hypermethylation	5'UTR

**14**	*DCR1 *18.19.20 (+183,+186,+189)	0.000001	**0.00002**	Hypermethylation	5'UTR

**15**	*DCR1 *22.23.24 (+231,+234,+237)	0.0001	**0.001**	Hypermethylation	Ex1 coding

**16**	*DCR1 *25.26.27 (+243,+246,+248)	0.0003	**0.002**	Hypermethylation	Ex1 coding

**17**	*DCR1 *32.33 (+306,+309)	0.00002	**0.0002**	Hypermethylation	Int 1-2

**18**	*DCR1 *34 (+327)	0.009	**0.041**	Hypermethylation	Int 1-2

**19**	*DCR2 *1 (+169)	0.0000007	**0.00001**	Hypermethylation	Ex1 coding

**20**	*DCR2 *2 (+166)	0.0000006	**0.00001**	Hypermethylation	Ex1 coding

**21**	*DCR2 *7.8 (+122,+118)	0.0000000007	**0.00000005**	Hypermethylation	Ex1 coding

**22**	*DCR2 *12.13 (+44,+38)	0.0000000003	**0.00000004**	Hypermethylation	5'UTR

**23**	*DCR2 *16 (+11)	0.0000001	**0.000003**	Hypermethylation	5'UTR

**24**	*DCR2 *17.18 (-14,-24)	0.0000002	**0.000006**	Hypermethylation	Promoter

**25**	*DCR2 *19 (-37)	0.0000003	**0.000007**	Hypermethylation	Promoter

**26**	*DCR2 *20 (-48)	0.000000002	**0.00000006**	Hypermethylation	Promoter

**27**	*DCR2 *24 (-178)	0.0000000008	**0.00000004**	Hypermethylation	Promoter

**28**	*CASP8 *2.3 (+532,+537)	0.000009	**0.0001**	Hypermethylation	Int 1-2

**29**	*CASP8 *6 (+635)	0.048	0.176	Hypomethylation	Int 1-3

**30**	*FLIP 18.19.20.21 *(+319,+326,+328,+332)	0.001	**0.008**	Hypomethylation	Int 1-4

**31**	*CYCS *17.18 (+59,+62)	0.001	**0.008**	Hypermethylation	5'UTR

**32**	*CYCS *19.20.21 (+89,+93,+99)	0.003	**0.016**	Hypermethylation	5'UTR

**33**	*TP53 *6 (-16)	0.017	0.067	Hypermethylation	Promoter

**34**	*BRCA1 *16 (-251)	0.005	**0.025**	Hypermethylation	Promoter

**35**	*BRCA1 *17 (-234)	0.016	0.065	Hypermethylation	Promoter

**36**	*BRCA2 *32.33 (-6,-8)	0.008	**0.038**	Hypermethylation	Promoter

**37**	*RNF8 *9.10.11.12 (-69,-67,-63,-58)	0.0001	**0.001**	Hypomethylation	Promoter

**38**	*H2AX *3 (-326)	0.002	**0.013**	Hypermethylation	Promoter

CpG site positions are mentioned with respect to the transcription start site. P^a ^value for Wilcoxon signed-rank test between breast tumor and adjacent normal tissue. P^b ^value for Wilcoxon signed-rank test between breast tumor and adjacent normal tissue after Benjamini Hochberg correction - significant values shown in bold.

**Figure 3:**
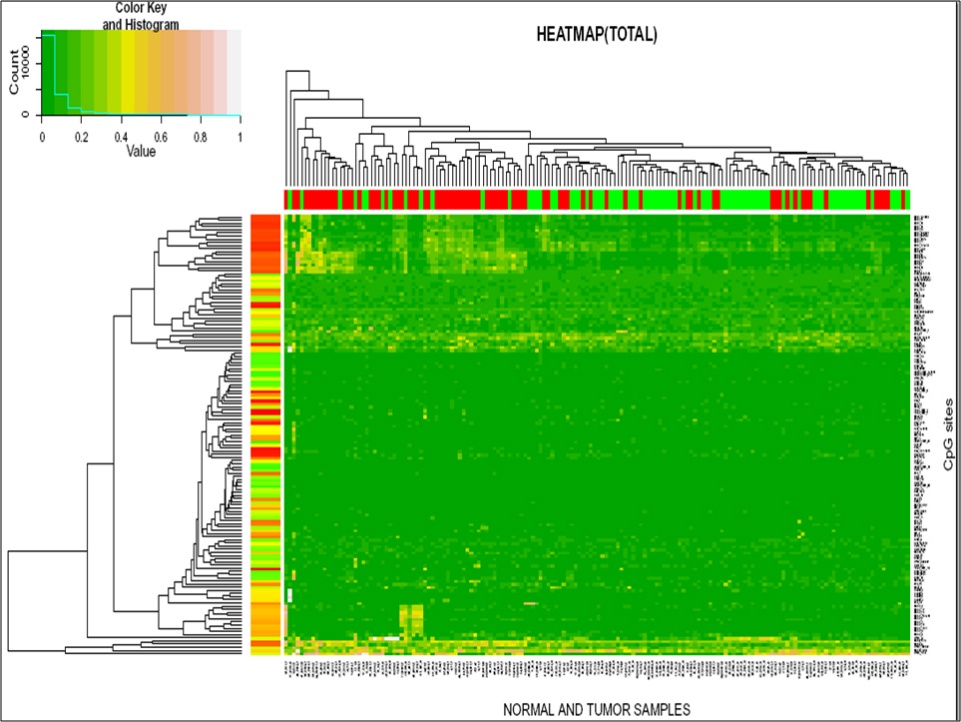
**Heatmap showing separate clustering of breast tumor (red) and normal (green) samples**.

Further, unsupervised two-way hierarchical clustering with 139 CpG sites/units in 81 pair of samples showed separate grouping of normal and breast tumor samples with negligible intermixing (Figure 3) as confirmed by Fisher's exact test (P = 0.00000001) (Table 2). We again performed unsupervised two-way hierarchical clustering for the 33 CpG units that were found to be significant after supervised clustering in Wilcoxon test and Benjamini Hochberg correction. An overall low methylation was observed in the normal samples as compared to tumor samples (Additional file 4, Figure S1).

**Table 2:** Fisher's exact test for validation of the presence of distinct tumor and normal groups based on methylation status of the DNA damage response and death receptor apoptotic pathway genes

Tumor/Normal group	Tumor (N = 81)	Normal (N = 81)	P^a^
**Tumor group (N = 62)**	49 (79.03%)	13 (20.96%)	

**Normal group (N = 100)**	32 (32.00%)	68 (68.00%)	0.00000001

P^a ^value for Fisher's exact test

**Figure 4:**
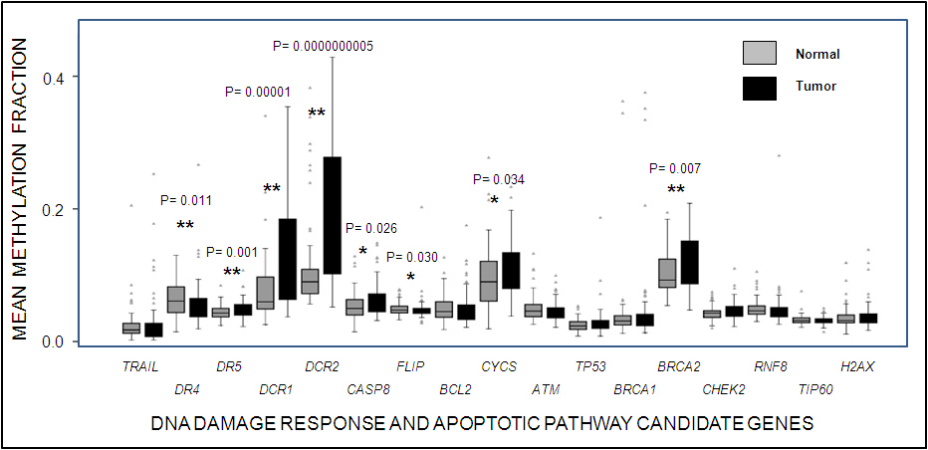
**Box plot showing mean methylation pattern between normal and tumor samples for the 17 genes studied**. Bottom and top of the box are the 25^th ^and 75^th ^percentile (the lower and upper quartiles, respectively), and the band near the middle of the box is the 50^th ^percentile (the median). The ends of the whiskers represents 5^th ^and 95^th ^percentile whereas data that are not included within these whiskers are plotted as an outlier (dot). * represent significant difference between normal and adjacent tumor samples. ** represent significant difference between normal and adjacent tumor samples after Benjamini Hochberg correction.

Mean methylation values (sample-wise average of methylation values of all CpG sites in one gene) reflected that *DR5, DCR1, DCR2 *and *BRCA2 *were hypermethylated whereas *DR4 *was hypomethylated in tumors as compared to adjacent normal tissues (Figure 4). Genes that did not show any difference between mean methylation pattern of normal and tumor tissues were: *TRAIL, BCL2, CASP8, CYCS, FLIP, ATM, TP53, CHEK2, RNF8, TIP60, H2AX *and *BRCA1*.

### Clinicopathological association

In order to understand the role of methylation in tumor progression, we compared the differential methylation pattern observed in normal versus tumor samples with respect to the various clinicopathological parameters (Table 3). ER negative tumors showed hypermethylation of DCR2 (87%; P = 0.00007) and hypomethylation of DR4 (77%; P = 0.001). Hypermethylation of DCR2 (75%; p = 0.001) in PR positive cases, hypomethylated DR4 (79%; p = 0.0002), hypermethylated DCR1 (64%; P = 0.004) and DCR2 (85%; P = 0.00003) in PR negative tumors was observed even after Benjamini Hochberg correction. Node negative tumors showed hypermethylation of DCR1 (63%; P = 0.003) and DCR2 (83%; P = 0.00001) while node positive displayed hypermethylation of DCR1 (66%, P = 0.001), DCR2 (73%; P = 0.00001) and BRCA2 (63%; P = 0.001). Collectively, tumor size 1 and 2 showed hypermethylation of DR5 (68%; P = 0.002), DCR1 (63%; P = 0.001) and DCR2 (80%; P = 0.0000001) as well as hypomethylation of DR4 (58%; P = 0.011), FLIP (61%; P = 0.007) and ATM (59%; P = 0.013) after Benjamini Hochberg correction. Similarly, tumor size 3 and 4 in combination showed hypermethylation of DCR1 (68%; P = 0.002) and DCR2 (73%; P = 0.001). Together, stage I and II tumors displayed hypermethylation of DCR1 (61%; P = 0.003) and DCR2 (82%; P = 0.000001), although hypomethylation was observed in some of the candidates but lost after Benjamini Hochberg correction. Likewise, stage III and IV tumors jointly showed hypermethylation in DCR1 (70%; P = 0.001) and DCR2 (70%; P = 0.0002) and BRCA2 (57%; P = 0.005). Grade I and II tumors exhibited hypermethylation in DCR2 (78%; P = 0.0002) while grade III tumors displayed DR5 (64%; P = 0.006), DCR1 (68%; P = 0.007) and DCR2 (84%; P = 0.0001) hypermethylation, and hypomethylation of DR4 (84%; P = 0.0003) and RNF8 (72%; P = 0.010).

**Table 3:** Comparison of mean methylation between normal and tumor samples for the 17 genes studied with respect to the various clinicopathological parameters

CLINICOPATHOLOGICAL PARAMETERS	*TRAIL*	*DR4*	*DR5*	*DCR1*	*DCR2*	*CASP8*	*FLIP*	*BCL2*	*CYCS*
**ER+ (N = 27)**	13^a^, 14^b^, 0^c^	11, 15, 1	8, 16, 3	6, 21, 0	6, 20, 1	12, 15, 0	14, 11, 2	18, 9, 0	14, 13, 0

**PERCENTAGE (%)**	48^a^, 52^b^, 0^c^	41, 56, 4	30, 59, 11	22, 78, 0	22, 74, 4	44, 56, 0	52, 41, 7	67, 33, 0	52, 48, 0

**P^a^**	0.857	0.741	0.137	0.007	0.004	0.791	0.115	0.095	0.81

**P^b^**	0.911	1.050	0.388	0.060	0.068	0.961	0.391	0.404	0.918

**ER- (N = 30)**	11, 14, 5	23, 7, 0	10, 20, 0	12, 18, 0	4, 26, 0	10, 18, 2	17, 13, 0	10, 20, 0	11, 19, 0

**PERCENTAGE (%)**	37, 47, 17	77, 23, 0	33, 67, 0	40, 60, 0	13, 87, 0	33, 60, 7	57, 43, 0	33, 67, 0	37, 63, 0

**P^a^**	0.228	0.001	0.010	0.013	0.00007	0.057	0.41	0.026	0.053

**P^b^**	0.298	**0.009**	0.057	0.055	**0.001**	0.108	0.498	0.074	0.113

**PR+ (N = 24)**	11, 13, 0	8, 15, 1	7, 16, 1	6, 18, 0	6, 18, 0	11, 13, 0	13, 9, 2	16, 8, 0	11, 13, 0

**PERCENTAGE (%)**	46, 54, 0	33, 63, 4	29, 67, 4	25, 75, 0	25, 75, 0	46, 54, 0	54, 38, 8	67, 33, 0	46, 54, 0

**P^a^**	0.989	0.196	0.063	0.015	0.001	0.977	0.123	0.157	0.310

**P^b^**	0.989	0.370	0.268	0.128	**0.017**	1.038	0.418	0.445	0.479

**PR- (N = 33)**	13, 15, 5	26, 7, 0	11, 20, 2	12, 21, 0	4, 28, 1	11, 20, 2	18, 15, 0	12, 21, 0	14, 19, 0

**PERCENTAGE (%)**	39, 45, 15	79, 21, 0	33, 61, 6	36, 64, 0	12, 85, 3	33, 61, 6	55, 45, 0	36, 64, 0	42, 58, 0

**P^a^**	0.36	0.0002	0.019	0.004	0.00003	0.082	0.325	0.098	0.180

**P^b^**	0.437	**0.002**	0.081	**0.023**	**0.001**	0.232	0.425	0.238	0.306

**NODE- (N = 40)**	19, 20, 1	20, 18, 2	11, 27, 2	15, 25, 0	6, 33, 1	18, 21, 1	25, 14, 1	20, 19, 1	15, 25, 0

**PERCENTAGE (%)**	48, 50, 3	50, 45, 5	28, 68, 5	38, 63, 0	15, 83, 3	45, 53, 3	63, 35, 3	50, 48, 3	36, 63, 0

**P^a^**	0.828	0.162	0.011	0.003	0.00001	0.267	0.107	0.581	0.021

**P^b^**	0.828	0.275	0.062	**0.026**	**0.0002**	0.378	0.202	0.658	0.071

**NODE+ (N = 41)**	21, 16, 4	27, 14, 0	14, 25, 2	14, 27, 0	10, 30, 1	13, 26, 2	20, 19, 2	21, 20, 0	18, 23, 0

**PERCENTAGE (%)**	51, 39, 10	66, 34, 0	34, 61, 5	34, 66, 05	24, 73, 2	32, 63, 5	49, 46, 5	51, 49, 0	44, 56, 0

**P^a^**	0.276	0.017	0.039	0.001	0.00001	0.032	0.165	0.564	0.496

**P^b^**	0.469	0.072	0.095	**0.006**	**0.0002**	0.091	0.312	0.685	0.703

**T1 + T2 (N = 59)**	33, 23, 3	34, 23, 2	16, 40, 3	22, 37, 0	11, 47, 1	23, 34, 4	36, 20, 3	33, 26, 0	27, 32, 0

**PERCENTAGE (%)**	56, 39, 5	58, 39, 3	27, 68, 5	37, 63, 0	19, 80, 2	39, 58, 3	61, 34, 5	56, 44, 0	46, 54, 0

**P^a^**	0.185	0.011	0.002	0.001	0.0000001	0.068	0.007	0.213	0.077

**P^b^**	0.262	**0.037**	**0.011**	**0.009**	**0.000002**	0.116	**0.030**	0.279	0.119

**T3+T4 (N = 22)**	7, 13, 2	13, 9, 0	9, 12, 1	7, 15, 0	5, 16, 1	8, 13, 1	9, 13, 0	8, 13, 1	6, 16, 0

**PERCENTAGE (%)**	32, 59, 9	59, 41, 0	41, 55, 5	32, 68, 0	23, 73, 5	36, 59, 5	41, 59, 0	36, 59, 5	27, 73, 0

**P^a^**	0.321	0.495	0.204	0.002	0.001	0.217	0.884	0.073	0.194

**P^b^**	0.496	0.647	0.495	**0.017**	**0.017**	0.461	0.884	0.310	0.550

**STAGE 1+2 (N = 51)**	25, 23, 3	27, 22, 2	15, 34, 2	20, 31, 0	9, 42, 0	21, 28, 2	31, 18, 2	28, 23, 0	22, 29, 0

**PERCENTAGE (%)**	49, 45, 6	53, 43, 4	29, 67, 4	39, 61, 0	18, 82, 0	41, 55, 4	61, 35, 4	55, 45, 0	43, 57, 0

**P^a^**	0.692	0.032	0.011	0.003	0.000001	0.118	0.035	0.436	0.074

**P^b^**	0.692	0.078	0.062	**0.026**	**0.00001**	0.182	0.074	0.570	0.140

**STAGE 3+4 (N = 30)**	15, 13, 2	20, 10, 0	10, 18, 2	9, 21, 0	7, 21, 2	10, 19, 1	14, 15, 1	13, 16, 1	11, 19, 0

**PERCENTAGE (%)**	50, 43, 7	67, 33, 0	33, 60, 7	30, 70, 0	23, 70, 7	33, 63, 3	47, 50, 3	43, 53, 3	37, 63, 0

**P^a^**	0.576	0.181	0.041	0.001	0.0002	0.100	0.347	0.279	0.217

**P^b^**	0.653	0.513	0.174	**0.009**	**0.003**	0.340	0.492	0.527	0.527

**GRADE 1+2 (N = 32)**	11, 19, 2	13, 18, 1	11, 20, 1	10, 22, 0	7, 25, 0	15, 17, 0	17, 13, 2	14, 18, 0	15, 17, 0

**PERCENTAGE (%)**	34, 59, 6	41, 56, 3	34, 63, 35	31, 69, 0	22, 78, 0	47, 53, 0	53, 41, 6	44, 56, 0	47, 53, 0

**P^a^**	0.085	0.681	0.088	0.009	0.0002	0.793	0.153	0.575	0.278

**P^b^**	0.361	0.827	0.299	0.077	**0.003**	0.899	0.434	0.815	0.675

**GRADE 3 (N = 25)**	13, 9, 3	21, 4, 0	7, 16, 2	8, 17, 0	3, 21, 1	7, 16, 2	14, 11, 0	14, 11, 0	10, 15, 0

**PERCENTAGE (%)**	52, 36, 12	84, 16, 0	28, 64, 8	32, 68, 0	12, 84, 4	28, 64, 8	56, 44, 0	56, 44, 0	40, 60, 0

**P^a^**	0.353	0.0003	0.006	0.007	0.0001	0.100	0.313	0.893	0.187

**P^b^**	0.500	**0.003**	**0.034**	**0.030**	**0.002**	0.283	0.484	0.893	0.318

**CLINICOPATHOLOGICAL PARAMETERS**	***ATM***	***TP53***	***BRCA1***	***BRCA2***	***CHEK2***	***TIP60***	***RNF8***	***H2AX***	

**ER+ (N = 27)**	16, 11, 0	14, 10, 3	10, 17, 0	9, 14, 4	13, 10, 4	12, 14, 1	18, 9, 0	13, 11, 3	

**PERCENTAGE (%)**	59, 41, 0	52, 37, 11	37, 63, 0	33, 52, 15	48, 37, 15	44, 52, 4	67, 33, 0	48, 41, 11	

**P^a^**	0.264	0.390	0.374	0.260	0.988	0.760	0.062	0.474	

**P^b^**	0.561	0.663	0.706	0.631	0.988	0.994	0.351	0.733	

**ER- (N = 30)**	12, 15, 3	8, 21, 1	14, 16, 0	7, 21, 2	14, 16, 0	16, 8, 6	17, 11, 2	11, 18, 1	

**PERCENTAGE (%)**	40, 50, 10	27, 70, 3	47, 53, 0	23, 70, 7	47, 53, 0	53, 27, 20	57, 37, 7	37, 60, 3	

**P^a^**	0.857	0.039	0.765	0.016	0.885	0.111	0.206	0.150	

**P^b^**	0.911	0.095	0.867	0.054	0.885	0.189	0.292	0.232	

**PR+ (N = 24)**	13, 11, 0	12, 9, 3	7, 17, 0	8, 13, 3	12, 10, 2	10, 12, 2	17, 7, 0	13, 9, 2	

**PERCENTAGE (%)**	54, 46, 0	50, 38, 13	29, 71, 0	33, 54, 13	50, 42, 8	42, 50, 8	71, 29, 0	54, 38, 8	

**P^a^**	0.658	0.519	0.174	0.192	0.974	0.858	0.039	0.298	

**P^b^**	0.860	0.735	0.423	0.408	1.104	1.042	0.221	0.507	

**PR- (N = 33)**	15, 15, 3	10, 22, 1	17, 16, 0	8, 22, 3	15, 16, 2	18, 10, 5	18, 13, 2	11, 20, 2	

**PERCENTAGE (%)**	45, 45, 9	30, 67, 3	52, 48, 0	24, 67, 9	45, 48, 6	55, 30, 15	55, 39, 6	33, 61, 6	

**P^a^**	0.53	0.120	0.907	0.023	0.814	0.230	0.243	0.141	

**P^b^**	0.601	0.255	0.907	0.078	0.865	0.355	0.344	0.266	

**NODE- (N = 40)**	22, 15, 3	13, 25, 2	14, 26, 0	14, 22, 4	18, 20, 2	14, 22, 4	22, 17, 1	13, 25, 2	

**PERCENTAGE (%)**	55, 38, 8	33, 63, 5	35, 65, 0	35, 55, 10	45, 50, 5	35, 55, 10	55, 43, 3	33, 63, 5	

**P^a^**	0.068	0.028	0.080	0.370	0.591	0.183	0.291	0.021	

**P^b^**	0.165	0.079	0.170	0.449	0.628	0.283	0.381	0.089	

**NODE+ (N = 41)**	21, 19, 1	19, 20, 2	20, 21, 0	10, 26, 5	17, 19, 5	27, 11, 3	24, 16, 1	17, 21, 3	

**PERCENTAGE (%)**	51, 46, 2	46, 49, 5	49, 51	24, 63, 12	41, 46, 12	66, 27, 7	59, 39, 2	41, 51, 7	

**P^a^**	0.973	0.553	0.979	0.001	0.465	0.031	0.120	0.942	

**P^b^**	1.034	0.723	0.979	**0.009**	0.719	0.105	0.255	1.068	

**T1 + T2 (N = 59)**	35, 21, 3	23, 33, 3	26, 33, 0	19, 36, 4	27, 28, 4	32, 24, 3	35, 23, 1	19, 37, 3	

**PERCENTAGE (%)**	59, 36, 5	39, 56, 5	44, 56, 0	32, 61, 7	46, 47, 7	54, 41, 5	59, 39, 2	32, 63, 5	

**P^a^**	0.013	0.407	0.694	0.056	0.639	0.276	0.037	0.040	

**P^b^**	**0.037**	0.461	0.694	0.106	0.679	0.335	0.090	0.085	

**T3+T4 (N = 22)**	8, 13, 1	9, 12, 1	8, 14, 0	5, 12, 5	8, 11, 3	9, 9, 4	11. 10, 1	11, 9, 2	

**PERCENTAGE (%)**	36, 59, 1	41, 55, 5	36, 64, 0	23, 55, 23	36, 50, 14	41, 41, 18	50, 45, 5	50, 41, 9	

**P^a^**	0.230	0.433	0.157	0.035	0.295	0.744	0.767	0.562	

**P^b^**	0.434	0.613	0.534	0.198	0.502	0.843	0.815	0.682	

**STAGE 1+2 (N = 51)**	31, 17, 3	20, 27, 4	21, 30, 0	18, 31, 2	23, 25, 3	25, 23, 3	32, 18, 1	17, 32, 2	

**PERCENTAGE (%)**	61, 33, 6	39, 53, 8	41, 59, 0	35, 61, 4	45, 49, 6	49, 45, 6	63, 35, 2	33, 63, 4	

**P^a^**	0.013	0.397	0.499	0.100	0.674	0.636	0.015	0.020	

**P^b^**	0.055	0.562	0.606	0.170	0.716	0.721	0.051	0.057	

**STAGE 3+4 (N = 30)**	12, 17, 1	12, 18, 0	13, 17, 0	6, 17, 7	12, 14, 4	16, 10, 4	14, 15, 1	13, 14, 3	

**PERCENTAGE (%)**	40, 57, 3	40, 60, 0	43, 57, 0	20, 57, 23	40, 47, 13	53, 33, 13	47, 50, 3	43, 47, 10	

**P^a^**	0.252	0.434	0.280	0.005	0.303	0.524	0.983	0.674	

**P^b^**	0.536	0.568	0.476	**0.028**	0.468	0.636	0.983	0.716	

**GRADE 1+2 (N = 32)**	13, 17, 2	14, 17, 1	11, 21, 0	9, 19, 4	13, 17, 2	14, 14, 4	17, 13, 2	16, 13, 3	

**PERCENTAGE (%)**	41, 53, 6	44, 53, 3	34, 66, 0	28, 59, 13	41, 53, 6	44, 44, 13	53, 41, 6	50, 41, 9	

**P^a^**	0.845	0.486	0.64	0.043	0.491	0.452	0.487	0.795	

**P^b^**	0.845	0.918	0.837	0.244	0.759	0.961	0.828	0.845	

**GRADE 3 (N = 25)**	15, 9, 1	8, 14, 3	13, 12, 0	7, 16, 2	14, 9, 2	14, 8, 3	18, 7, 0	8, 16,1	

**PERCENTAGE (%)**	60, 36, 4	32, 56, 12	52, 48, 0	28, 64, 8	16, 76, 8	56, 32, 12	72, 28, 0	32, 64, 4	

**P^a^**	0.141	0.494	0.686	0.114	0.523	0.648	0.010	0.121	

**P^b^**	0.266	0.646	0.729	0.277	0.635	0.734	**0.034**	0.257	

P^a ^value for Wilcoxon signed-rank test between breast tumor and adjacent normal tissue. P^b ^value for Wilcoxon signed-rank test between breast tumor and adjacent normal tissue after Benjamini Hochberg correction - significant values shown in bold. a: Number and corresponding percentage of hypomethylated tumors compared to adjacent normal, b: Number and corresponding percentage of hypermethylated tumors compared to adjacent normal, c: Number and corresponding percentage of tumors that show same methylation compared to adjacent normal.

### Real time expression analysis of DDR - apoptotic pathway genes and their correlation with the methylation pattern

**Figure 5:**
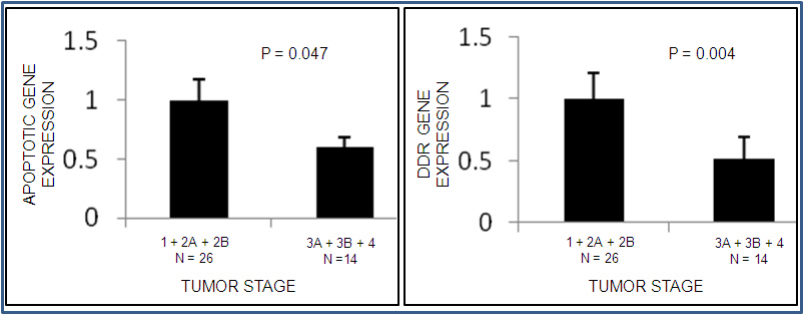
**Categorization of expression pattern of genes involved in death receptor apoptotic pathway [(*TRAIL + DR4 + DR5 + CASP8 + ATM + H2AX + CHEK2 + CYCS)/(DCR1 + DCR2 + CASP8L + FLIPL + FLIPS + BCL2*)] and DNA damage response pathway [*ATM + CHEK2 + BRCA1 + BRCA2 + H2AX*], stratified with respect to tumor stage**.

In order to understand the level of DNA damage repair and apoptosis with respect to tumor progression, a representative set of 40 tumor samples were studied for the real time expression of 15 genes (*TRAIL, DR4, DR5, DCR1, DCR2, CASP8, CASP8L, FLIPL, FLIPS, BCL2, CYCS, ATM, TP53, BRCA1, BRCA2, CHEK2 *and *H2AX*) belonging to the DDR and apoptotic pathway. The results of expression for these genes were categorized in pro- apoptotic versus anti- apoptotic signals and DNA damage response signals with respect to the tumor stages. The results showed that DNA damage response as well as apoptosis decreased with advancing sporadic breast tumors (P = 0.004; 0.047) (Figure 5) (Additional file 5, Figure S2).

Also, the relationship between promoter methylation and gene expression was evaluated. Spearman's correlation coefficient was calculated for each CpG unit. Negative correlation was observed for *TRAIL *-116 (P = 0.013; ρ = -0.350), *DR4 *+108 (P = 0.025; ρ = -0.331), *CASP8 *+571 (P = 0.046; ρ = -0.271), *ATM *-369 (P = 0.046; ρ = -0.270), *ATM *-314, -310 (P = 0.034; ρ = -0.291), *CHEK2 *-396, -393 (P = 0.040; ρ = -0.281), *BRCA1 *-314 (P = 0.030; ρ = -0.300), *BRCA1 *-251 (P = 0.004; ρ = -0.410), *BRCA2 *-101 (P = 0.036; ρ = -0.288) and *BRCA2 *+133, +136, +138 (P = 0.023; ρ = -0.318). However, positive correlation was observed for *DR4 *+23, +25, +28, +32 (P = 0.017; ρ = 0.335) and *CYCS *+241, +243 (P = 0.048; ρ = 0.266) (Table 4). On other hand, no correlation was observed for *DR5, FLIP, BCL2, TP53 *and *H2AX. Insilico *analysis using Alibaba2.1 revealed the presence of Sp1, ER and GBF2 transcription factor (TF) binding sites or absence of TF consensus sequence at these positions.

**Table 4:** Correlation of methylation of tumor samples with respect to the transcript expression

SR. NO.	GENE	CpG SITE/UNIT (N = 40)	SPEARMAN'S RHO (ρ) CORRELATION COEFFICIENT	P^a^	LOCATION	TRANSCRIPTION FACTOR (TF) BINDING SITE
1	*TRAIL*	*TRAIL *9 (-116)	-0.350	0.013	Promoter	No

2	*DR4*	*DR4 *10.11.12.13 (+23,+25,+28,+32)	0.335	0.017	5'UTR	GBF2

3	*DR4*	*DR4 *7 (+108)	-0.311	0.025	5'UTR	No

4	*CASP8*	*CASP8 *5 (+571)	-0.271	0.046	Int 1-2	No

5	*CYCS*	*CYCS *36.37 (+241,+243)	0.266	0.048	Int 1-2	Sp1

6	*ATM*	*ATM *1 (-369)	-0.270	0.046	Promoter	No

7	*ATM*	*ATM *7.8 (-314,-310)	-0.291	0.034	Promoter	No

8	*CHEK2*	*CHEK2 *3.4 (-396,-393)	-0.281	0.040	Promoter	Sp1

9	*BRCA1*	*BRCA1 *8 (-314)	-0.300	0.030	Promoter	No

10	*BRCA1*	*BRCA1 *16 (-251)	-0.410	0.004	Promoter	No

11	*BRCA2*	*BRCA2 *44 (-101)	-0.288	0.036	Promoter	Sp1

12	*BRCA2*	*BRCA2 *15.16.17 (+133,+136,+138)	-0.318	0.023	5'UTR	ER, Sp1

P^a ^value for Spearman's rho correlation coefficient.

### Involvement of *BRCA2 *in sporadic breast tumor

**Figure 6:**
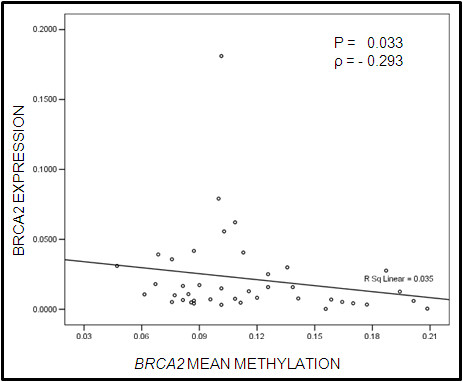
**Comparison of mean methylation of *BRCA2 *gene with its transcript expression (ρ: spearman's correlation coefficient)**.

Earlier studies from our laboratory have identified *BRCA2 *-26 wild type GG and mutant AA genotype to provide risk whereas heterozygote GA to provide protection against sporadic breast tumors [6]. Categorization of breast tumor samples on the basis of BRCA2 -26 G/A 5'UTR polymorphism, followed by methylation status of the candidate genes resulted in the identification of GG and AA genotypes to be associated with hypermethylated *BRCA2 *(63%;P = 0.008) *, DR5 *(69%;P = 0.001)*, DCR1 *(67%; P = 0.0001)*, DCR2 *(76%;P = 0.0000002)*, CASP8 *(59%;P = 0.006) and hypomethylated *FLIP *(59%;P = 0.009). Whereas, the GA protector genotype was found to be associated with hypermethylated *DCR2 *(81%; P = 0.0008) (Table 5). Expression data for BRCA2 transcript compared with *BRCA2 *mean methylation level, revealed a negative correlation (P = 0.033; ρ = -293) (Figure 6).

**Table 5:** Comparison of mean methylation between normal and tumor samples for the 17 genes studied with respect to the BRCA2 -26 5'UTR polymorphism

GENES	BRCA2 -26 GG + AA (N = 54)				BRCA2 -26 GA (N = 27)			
	METHYLATION	PERCENTAGE	P^a^	P^b^	METHYLATION	PERCENTAGE	P^a^	P^b^
*TRAIL*	26 ^a^, 26 ^b^, 2 ^c^	48% ^a^, 48% ^b^, 4% ^c^	0.655	0.696	14, 10, 3	52%, 37%, 11%	0.555	0.858

*DR4*	31, 21, 2	57%, 39%, 4%	0.033	0.070	16, 11, 0	59%, 41%, 0%	0.175	0.595

*DR5*	14, 37, 3	26%, 69%, 6%	0.001	**0.006**	11, 15, 1	41%, 56%, 4%	0.394	0.837

*DCR1*	18, 36, 0	33%, 67%, 0%	0.0001	**0.001**	11, 16, 0	41%, 59%, 0%	0.038	0.215

*DCR2*	12, 41, 1	22%, 76%, 2%	0.0000002	**0.000003**	4, 22, 1	15%, 81%, 4%	0.0008	**0.014**

*CASP8*	20, 32, 2	37%, 59%, 4%	0.006	**0.026**	11, 15, 1	41%, 56%, 4%	0.970	0.970

*BCL2*	29, 24, 1	54%, 44%, 2%	0.972	0.972	12, 15, 0	44%, 56%, 0%	0.914	1.036

*FLIP*	32, 19, 3	59%, 35%, 6%	0.009	**0.026**	13, 14, 0	48%, 52%, 0%	0.952	1.012

*CYCS*	25, 29, 0	46%, 54%, 0%	0.164	0.253	8, 19, 0	30%, 70%, 0%	0.139	0.591

*ATM*	30, 22, 2	56%, 41%, 4%	0.303	0.429	13, 12, 2	48%, 44%, 7%	0.443	0.837

*TP53*	18, 33, 3	33%, 61%, 6%	0.029	0.070	14, 12, 1	52%, 44%, 4%	0.334	0.811

*BRCA1*	19, 35, 0	35%, 65%, 0%	0.065	0.123	15, 12, 0	56%, 44%, 0%	0.648	0.918

*BRCA2*	15, 34, 5	28%, 63%, 9%	0.008	**0.027**	9, 14, 4	33%, 52%, 15%	0.273	0.774

*CHEK2*	23, 25, 6	43%, 46%, 11%	0.320	0.418	12, 14, 1	44%, 52%, 4%	0.829	1.084

*TIP60*	28, 21, 5	52%, 39%, 9%	0.466	0.528	13, 12, 2	48%, 44%, 7%	0.893	1.084

*RNF8*	28, 24, 2	52%, 44%, 4%	0.454	0.551	18, 9, 0	67%, 33%, 0%	0.025	0.213

*H2AX*	17, 34, 3	31%, 63%, 6%	0.152	0.258	13, 12, 2	48%, 44%, 7%	0.535	0.910

P^a ^value for Wilcoxon signed-rank test between breast tumor and adjacent normal tissue. P^b ^value for Wilcoxon signed-rank test between breast tumor and adjacent normal tissue after Benjamini Hochberg correction - significant values shown in bold. a: Number and corresponding percentage of hypomethylated tumors compared to adjacent normal, b: Number and corresponding percentage of hypermethylated tumors compared to adjacent normal, c: Number and corresponding percentage of tumors that show same methylation compared to adjacent normal.

## Discussion

Methylation alterations in cancer have been recognized for decades [47-50]. We analyzed quantitative changes in methylation of 17 promoter regions in 162 paired normal and cancerous breast tissues from 81 sporadic breast cancer patients using high throughput MALDI-TOF MS and evaluated their distribution, correlation and relationships to clinicopathological variables using common statistical methods. The genes selected belonged to the DNA damage response (DDR) and death receptor apoptotic pathways [30,51-54]. Among the studied 17 genes, this is the first report of promoter methylation for CpG sites in *H2AX, RNF8 *and *CYCS *in human cancer. The limitation of the study was our ability to score only 46.14% of the total CpG of the studied region and the absence of screening of CpG site methylation for the entire promoter and the 5'UTR.

Although methylation levels observed in breast tissues were not very high, yet a significantly differential methylation pattern existed between normal and tumor samples, suggesting that the epigenetic control of the gene promoters was required for the maintenance of normal cellular homeostasis, deregulation of which could result in tumor development. Based on the DNA damage response and apoptotic pathway gene methylation profile, we were able to segregate the normal and tumor tissues. Our observation followed by statistical analysis of methylation status in breast tumors along with transcript expression revealed a negative correlation for *TRAIL, DR4, CASP8, ATM, CHEK2*, *BRCA1 *and *BRCA2 *CpG sites. *Insilico *analysis of these methylated CpG sites/units revealed the presence of consensus sequence for stimulatory protein 1 (Sp1) and estrogen receptor (ER) transcription factors. Studies have revealed that methylated CpGs in these recognition sites preclude the binding of the Sp1 transcription factor and thereby inhibit gene expression directly [55,56]. DNA methylation has also been shown to be followed by binding of methyl-CpG binding domain proteins (MBDs) which contribute to gene repression by the recruitment of histone deacetylases (HDACs) to nucleosomes [57]. The CpG sites within *CHEK2 *and *BRCA2 *were observed to be the consensus sequence for Sp1; whereas such sites in *TRAIL, DR4, CASP8, ATM *and *BRCA1 *did not have transcription factor binding sites. We believe methylation of these sites might inhibit/promote the binding of Sp1/MBDs, respectively to the methylated DNA, resulting in decreased gene expression.

For the first time, our study showed a collaborative involvement of *DR5, DCR1, DCR2, CASP8, CYCS, BRCA1, BRCA2, H2AX *hypermethylation and *DR4, FLIP, RNF8 *hypomethylation in sporadic breast tumor pathogenesis. Since apoptotic signaling through DR5 has been reported to be more potent than through DR4 [58], our data suggests that hypermethylation status of *DR5 *receptor facilitates tumor cells to evade apoptosis. Despite the hypomethylation state of DR4 receptor and hypermethylation state of anti-apoptotic decoy receptors (DCR1 and DCR2) [25,42,43], we propose that the final outcome in tumor pathogenesis depends on downstream signal transduction molecules, such as *CASP8, FLIP *and *CYCS *in established tumors. Hypermethylation of *CASP8 *and hypomethylation of *FLIP *inhibits the extrinsic apoptotic pathway, whereas hypermethylation of *CYCS *suggests a weak activation of intrinsic apoptotic pathway. Similarly, hypermethylation of *BRCA1, BRCA2 *and *H2AX *implies a decreased DNA damage repair. This is in accordance with the knowledge that inhibition of DNA damage response and apoptosis may contribute to tumor initiation, growth and metastasis in the pathogenesis of breast cancer [59,60].

**Figure 7:**
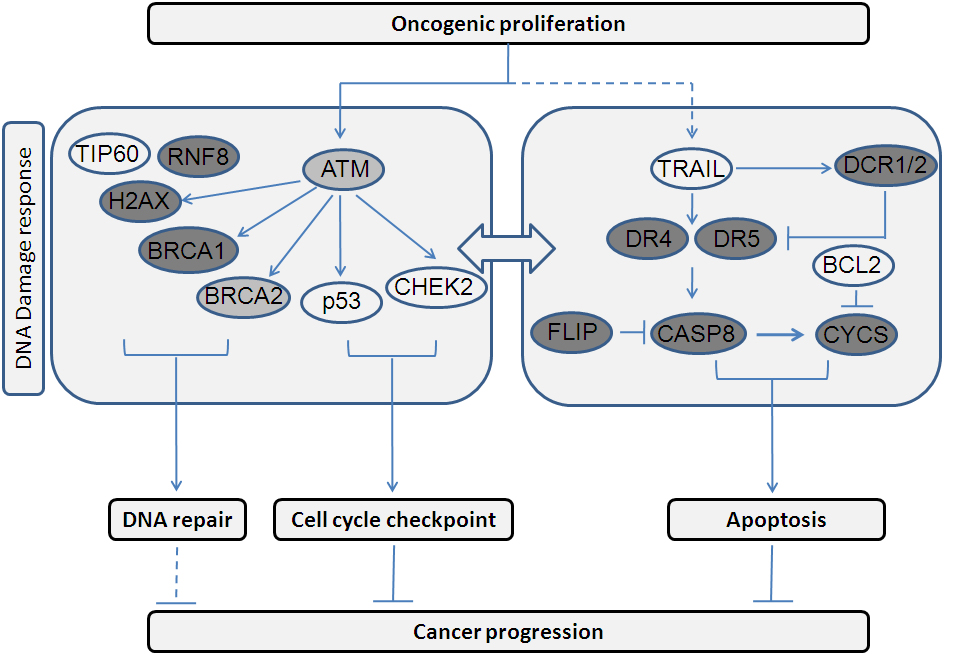
**Model depicting the role of various DNA damage response (DDR) - apoptotic genes in epigenetic control of tumorigenesis**. Dark grey oval represents candidate genes that play a major role across all tumor subtypes. Light grey oval represents candidate genes responsible for either initiation or progression of tumor.

We believe decoy receptor down-regulation through hypermethylation in initial (stages I and II) as well as advanced stages (stage III and IV) represents a 'physiological' response to check the conversion of precancerous cell to a cancerous one by enhancing apoptosis, which can be evaded by established tumors via mechanisms such as aberrant methylation of DDR and apoptotic genes. The methylation profiles of the candidate genes in this pathway revealed increased apoptosis in initial tumors (T1 and T2) as a result of hypomethylated *DR4 *which initiates the death receptor signal transduction pathway in response to TRAIL. Although in initial tumors (T1 and T2) hypermethylation of DR5 and hypomethylation of *FLIP *is observed, we believe, the positive feedback loop due to hypomethylated *ATM *is strong enough to override the antiapoptotic effect of *FLIP *thereby enhancing DR4 mediated apoptosis. However, in advanced tumors (T3 and T4) most of the methylation profiles observed in initial tumor stages are lost consequently, resulting in decreased apoptosis. Hypermethylation of DR5 in grade III tumors also suggests a reduced apoptotic potential in more aggressive form of breast tumors. In the context of TRAIL mediating the activation of DNA damage response pathway [28,29], hypermethylation of *BRCA2 *(stage III and IV) indicated an aberrant DNA damage repair mechanism in response to TRAIL mediated apoptosis. Thus our study showed a deregulation of death receptor apoptosis and DDR pathway genes through methylation, influencing breast tumorigenesis; also indicated by reduced apoptosis and DNA repair in advanced tumor. This is in accordance with the fact that suppression of apoptosis and DNA damage repair comprise the minimum common platform upon which all neoplastic evolution occurs [61]. We suggest that besides genetic aberrations of DDR-apoptotic genes, their deregulated methylation also plays an intricate role in tumor development. On the whole, the study revealed cooperative involvement of the DDR and apoptotic genes at the level of methylation in assisting tumor cell survival and progression by apoptotic evasion and loss of DNA repair during sporadic breast tumor progression (Figure 7).

In addition, BRCA2 germline variation concomitant with the presence of methylation in the promoter region was novel and interesting and emerged as a strong candidate for susceptibility to sporadic breast tumors. Flanagan *et.al*. [21] showed in familial breast cancers that methylation profiles are defined by mutation status. On the basis of BRCA2 -26 GA heterozygotes observed earlier by us with a protector genotype, showing decreased LOH; and the risk providing GG/AA homozygotes showing increased LOH [6], we suggest that the presence of hypermethylated *DR5, CASP8, BRCA2 *and hypomethylated *FLIP *with *BRCA2 *-26 GG or AA genotype, might cause increased LOH, decreased DNA repair and apoptosis, providing a risk for sporadic breast tumor development and progression. These observations allow us to assign an important role to a concomitant presence of variant germline genetic background at 5'UTR of BRCA2 and the epigenetic modification in the process of oncogenesis.

No genome-wide evaluation have been carried out to identify altered DNA methylation patterns in the context of tumor initiation and/or progression [62] and their effects on DNA damage response - apoptotic pathway or gene networks. Our study indicates that promoter methylation of DDR-apoptotic genes in sporadic breast cancer is not a random phenomenon. It has two features: hypermethylation of DDR-apoptotic genes, and hypomethylation of anti-apoptotic/pro-survival genes. Progressive modification of the aberrant epigenetic alterations with advancing tumors results in deregulation of the DDR-apoptotic pathway thereby promoting tumor development. We also observe BRCA2 to play a major role at both genetic and epigenetic level in sporadic breast tumor pathogenesis. We propose, since pathological epigenetic changes of the DDR-apoptotic genes are reversible modifications [63], these could further be targeted for therapeutic interventions.

## List of abbreviations

ATM: Ataxia Telangiectasia Mutated; BCL2: B cell lymphoma 2; BRCA1/2: Breast Cancer 1 and 2, early onset proteins; CASP8: Caspase 8; CHEK2: Checkpoint Kinase 2; CYCS: Cytochrome c; DCR1/2: Decoy Receptors 1/2; DR4/5: Death Receptors 4/5; FLIPL/S: FLICE like inhibitory protein large/small; H2AX: Histone H2A-member X; RNF8: Ring finger protein 8; TIP60/KAT5: Lysine acetyltransferase 5; TRAIL: Tumor necrosis factor related apoptosis inducing ligand

## Competing interests

The authors declare that they have no competing interests.

## Authors' contributions

RP participated in the design of the experiments, experimental data acquisition, statistical analysis, interpretation of data and the writing of the draft. NS participated in the design of the experiments and interpretation of data. RC participated in experimental data acquisition. SG participated in the interpretation of data and gave critical suggestions. PG, NP and GA, as clinician and surgeon, participated in the acquisition of data. RB conceived the study, participated in the design of the experiments and the interpretation of data, revised the draft critically for intellectual content, and gave final approval of the version to be published. All authors have read and approved the final manuscript.

## Supplementary Material

Additional file 1 Table S1: Listing of Breast tumor samples studied for methylation status of the DDR - apoptotic genes Click here for file

Additional file 2 Table S2: Tagged methylation primers designed using epidesigner software Click here for file

Additional file 3 Table S3: CpG summary of the 17 genes belonging to the DDR-apoptotic pathway Click here for file

Additional file 4 Figure S1: Heatmap showing differential methylation pattern in breast tumor (red) and normal (green) Click here for file

Additional file 5 Figure S2: Categorization of expression pattern of individual death receptor apoptotic pathway and DNA damage response pathway genes stratified with respect to the breast tumor stage Click here for file

## References

[B1] Xu X Gammon MD Zhang Y Bestor TH Zeisel SH Wetmur JG Wallenstein S Bradshaw PT Garbowski G Teitelbaum SL BRCA1 promoter methylation is associated with increased mortality among women with breast cancer Breast Cancer Res Treat 2009 115 397 404 10.1007/s10549-008-0075-5 18521744 PMC2693263

[B2] Russo J Yang X Hu YF Bove BA Huang Y Silva ID Tahin Q Wu Y Higgy N Zekri A Russo IH Biological and molecular basis of human breast cancer Front Biosci 1998 3 D944 960 9727085 10.2741/a335

[B3] Feinberg AP Tycko B The history of cancer epigenetics Nat Rev Cancer 2004 4 143 153 10.1038/nrc1279 14732866

[B4] Baylin SB Ohm JE Epigenetic gene silencing in cancer - a mechanism for early oncogenic pathway addiction? Nat Rev Cancer 2006 6 107 116 10.1038/nrc1799 16491070

[B5] Oldenburg RA Meijers-Heijboer H Cornelisse CJ Devilee P Genetic susceptibility for breast cancer: how many more genes to be found? Crit Rev Oncol Hematol 2007 63 125 149 10.1016/j.critrevonc.2006.12.004 17498966

[B6] Gochhait S Bukhari SI Bairwa N Vadhera S Darvishi K Raish M Gupta P Husain SA Bamezai RN Implication of BRCA2 -26G > A 5' untranslated region polymorphism in susceptibility to sporadic breast cancer and its modulation by p53 codon 72 Arg > Pro polymorphism Breast Cancer Res 2007 9 R71 10.1186/bcr1780 17945002 PMC2242669

[B7] Saha A Dhir A Ranjan A Gupta V Bairwa N Bamezai R Functional IFNG polymorphism in intron 1 in association with an increased risk to promote sporadic breast cancer Immunogenetics 2005 57 165 171 10.1007/s00251-005-0783-5 15900487

[B8] Saha A Gupta V Bairwa NK Malhotra D Bamezai R Transforming growth factor-beta1 genotype in sporadic breast cancer patients from India: status of enhancer, promoter, 5'-untranslated-region and exon-1 polymorphisms Eur J Immunogenet 2004 31 37 42 10.1111/j.1365-2370.2004.00442.x 15009180

[B9] Pal R Gochhait S Chattopadhyay S Gupta P Prakash N Agarwal G Chaturvedi A Husain N Husain SA Bamezai RN Functional implication of TRAIL -716 C/T promoter polymorphism on its in vitro and in vivo expression and the susceptibility to sporadic breast tumor Breast Cancer Res Treat 2010 20443055 10.1007/s10549-010-0900-5

[B10] Darvishi K Sharma S Bhat AK Rai E Bamezai RN Mitochondrial DNA G10398A polymorphism imparts maternal Haplogroup N a risk for breast and esophageal cancer Cancer Lett 2007 249 249 255 10.1016/j.canlet.2006.09.005 17081685

[B11] Saha A Bairwa NK Ranjan A Gupta V Bamezai R Two novel somatic mutations in the human interleukin 6 promoter region in a patient with sporadic breast cancer Eur J Immunogenet 2003 30 397 400 10.1111/j.1365-2370.2003.00423.x 14675392

[B12] Gochhait S Bhatt A Sharma S Singh YP Gupta P Bamezai RN Concomitant presence of mutations in mitochondrial genome and p53 in cancer development - a study in north Indian sporadic breast and esophageal cancer patients Int J Cancer 2008 123 2580 2586 10.1002/ijc.23817 18792899

[B13] Srivastava N Gochhait S Gupta P Bamezai RN Copy number alterations of the H2AFX gene in sporadic breast cancer patients Cancer Genet Cytogenet 2008 180 121 128 10.1016/j.cancergencyto.2007.09.024 18206537

[B14] Gochhait S Dar S Pal R Gupta P Bamezai RN Expression of DNA damage response genes indicate progressive breast tumors Cancer Lett 2009 273 305 311 10.1016/j.canlet.2008.08.009 18805634

[B15] Veeck J Esteller M Breast cancer epigenetics: from DNA methylation to microRNAs J Mammary Gland Biol Neoplasia 2010 15 5 17 10.1007/s10911-010-9165-1 20101446 PMC2824126

[B16] Stanssens P Zabeau M Meersseman G Remes G Gansemans Y Storm N Hartmer R Honisch C Rodi CP Bocker S van den Boom D High-throughput MALDI-TOF discovery of genomic sequence polymorphisms Genome Res 2004 14 126 133 10.1101/gr.1692304 14707174 PMC314289

[B17] Ehrich M Nelson MR Stanssens P Zabeau M Liloglou T Xinarianos G Cantor CR Field JK van den Boom D Quantitative high-throughput analysis of DNA methylation patterns by base-specific cleavage and mass spectrometry Proc Natl Acad Sci USA 2005 102 15785 15790 10.1073/pnas.0507816102 16243968 PMC1276092

[B18] Ehrich M Turner J Gibbs P Lipton L Giovanneti M Cantor C van den Boom D Cytosine methylation profiling of cancer cell lines Proc Natl Acad Sci USA 2008 105 4844 4849 10.1073/pnas.0712251105 18353987 PMC2290817

[B19] Radpour R Kohler C Haghighi MM Fan AX Holzgreve W Zhong XY Methylation profiles of 22 candidate genes in breast cancer using high-throughput MALDI-TOF mass array Oncogene 2009 28 2969 2978 10.1038/onc.2009.149 19503099

[B20] Radpour R Haghighi MM Fan AX Torbati PM Hahn S Holzgreve W Zhong XY High-throughput hacking of the methylation patterns in breast cancer by in vitro transcription and thymidine-specific cleavage mass array on MALDI-TOF silico-chip Mol Cancer Res 2008 6 1702 1709 10.1158/1541-7786.MCR-08-0262 19010818

[B21] Flanagan JM Cocciardi S Waddell N Johnstone CN Marsh A Henderson S Simpson P da Silva L Khanna K Lakhani S DNA methylome of familial breast cancer identifies distinct profiles defined by mutation status Am J Hum Genet 2010 86 420 433 10.1016/j.ajhg.2010.02.008 20206335 PMC2833389

[B22] Ehrich M Field JK Liloglou T Xinarianos G Oeth P Nelson MR Cantor CR van den Boom D Cytosine methylation profiles as a molecular marker in non-small cell lung cancer Cancer Res 2006 66 10911 10918 10.1158/0008-5472.CAN-06-0400 17108128

[B23] Ordway JM Budiman MA Korshunova Y Maloney RK Bedell JA Citek RW Bacher B Peterson S Rohlfing T Hall J Identification of novel high-frequency DNA methylation changes in breast cancer PLoS One 2007 2 e1314 10.1371/journal.pone.0001314 18091988 PMC2117343

[B24] Holm K Hegardt C Staaf J Vallon-Christersson J Jonsson G Olsson H Borg A Ringner M Molecular subtypes of breast cancer are associated with characteristic DNA methylation patterns Breast Cancer Res 2010 12 R36 10.1186/bcr2590 20565864 PMC2917031

[B25] Johnstone RW Frew AJ Smyth MJ The TRAIL apoptotic pathway in cancer onset, progression and therapy Nat Rev Cancer 2008 8 782 798 10.1038/nrc2465 18813321

[B26] Falschlehner C Emmerich CH Gerlach B Walczak H TRAIL signalling: decisions between life and death Int J Biochem Cell Biol 2007 39 1462 1475 10.1016/j.biocel.2007.02.007 17403612

[B27] Ashkenazi A Directing cancer cells to self-destruct with pro-apoptotic receptor agonists Nat Rev Drug Discov 2008 7 1001 1012 10.1038/nrd2637 18989337

[B28] Solier S Pommier Y The apoptotic ring: a novel entity with phosphorylated histones H2AX and H2B and activated DNA damage response kinases Cell Cycle 2009 8 1853 1859 19448405 10.4161/cc.8.12.8865

[B29] Solier S Sordet O Kohn KW Pommier Y Death receptor-induced activation of the Chk2- and histone H2AX-associated DNA damage response pathways Mol Cell Biol 2009 29 68 82 10.1128/MCB.00581-08 18955500 PMC2612481

[B30] Harper JW Elledge SJ The DNA damage response: ten years after Mol Cell 2007 28 739 745 10.1016/j.molcel.2007.11.015 18082599

[B31] Meek DW Tumour suppression by p53: a role for the DNA damage response? Nat Rev Cancer 2009 9 714 723 19730431 10.1038/nrc2716

[B32] Ravi R Bedi A Sensitization of tumor cells to Apo2 ligand/TRAIL-induced apoptosis by inhibition of casein kinase II Cancer Res 2002 62 4180 4185 12154014

[B33] Ray S Shyam S Fraizer GC Almasan A S-phase checkpoints regulate Apo2 ligand/TRAIL and CPT-11-induced apoptosis of prostate cancer cells Mol Cancer Ther 2007 6 1368 1378 10.1158/1535-7163.MCT-05-0414 17431115

[B34] Liu X Yue P Khuri FR Sun SY p53 upregulates death receptor 4 expression through an intronic p53 binding site Cancer Res 2004 64 5078 5083 10.1158/0008-5472.CAN-04-1195 15289308

[B35] Takimoto R El-Deiry WS Wild-type p53 transactivates the KILLER/DR5 gene through an intronic sequence-specific DNA-binding site Oncogene 2000 19 1735 1743 10.1038/sj.onc.1203489 10777207

[B36] Kuribayashi K Krigsfeld G Wang W Xu J Mayes PA Dicker DT Wu GS El-Deiry WS TNFSF10 (TRAIL), a p53 target gene that mediates p53-dependent cell death Cancer Biol Ther 2008 7 2034 2038 19106633 10.4161/cbt.7.12.7460

[B37] Fukazawa T Fujiwara T Uno F Teraishi F Kadowaki Y Itoshima T Takata Y Kagawa S Roth JA Tschopp J Tanaka N Accelerated degradation of cellular FLIP protein through the ubiquitin-proteasome pathway in p53-mediated apoptosis of human cancer cells Oncogene 2001 20 5225 5231 10.1038/sj.onc.1204673 11526513

[B38] Zhivotovsky B Kroemer G Apoptosis and genomic instability Nat Rev Mol Cell Biol 2004 5 752 762 10.1038/nrm1443 15340382

[B39] Sykes SM Mellert HS Holbert MA Li K Marmorstein R Lane WS McMahon SB Acetylation of the p53 DNA-binding domain regulates apoptosis induction Mol Cell 2006 24 841 851 10.1016/j.molcel.2006.11.026 17189187 PMC1766330

[B40] Sancar A Lindsey-Boltz LA Unsal-Kacmaz K Linn S Molecular mechanisms of mammalian DNA repair and the DNA damage checkpoints Annu Rev Biochem 2004 73 39 85 10.1146/annurev.biochem.73.011303.073723 15189136

[B41] Yang Q Kiernan CM Tian Y Salwen HR Chlenski A Brumback BA London WB Cohn SL Methylation of CASP8, DCR2, and HIN-1 in neuroblastoma is associated with poor outcome Clin Cancer Res 2007 13 3191 3197 10.1158/1078-0432.CCR-06-2846 17545522

[B42] van Noesel MM van Bezouw S Salomons GS Voute PA Pieters R Baylin SB Herman JG Versteeg R Tumor-specific down-regulation of the tumor necrosis factor-related apoptosis-inducing ligand decoy receptors DcR1 and DcR2 is associated with dense promoter hypermethylation Cancer Res 2002 62 2157 2161 11929838

[B43] Shivapurkar N Toyooka S Toyooka KO Reddy J Miyajima K Suzuki M Shigematsu H Takahashi T Parikh G Pass HI Aberrant methylation of trail decoy receptor genes is frequent in multiple tumor types Int J Cancer 2004 109 786 792 10.1002/ijc.20041 14999791

[B44] Kang JH Kim SJ Noh DY Park IA Choe KJ Yoo OJ Kang HS Methylation in the p53 promoter is a supplementary route to breast carcinogenesis: correlation between CpG methylation in the p53 promoter and the mutation of the p53 gene in the progression from ductal carcinoma in situ to invasive ductal carcinoma Lab Invest 2001 81 573 579 11304577 10.1038/labinvest.3780266

[B45] Cucer N Taheri S Ok E Ozkul Y Methylation status of CpG islands at sites -59 to +96 in exon 1 of the BRCA2 gene varies in mammary tissue among women with sporadic breast cancer J Genet 2008 87 155 158 10.1007/s12041-008-0023-5 18776644

[B46] McShane LM Altman DG Sauerbrei W Taube SE Gion M Clark GM Reporting recommendations for tumor marker prognostic studies J Clin Oncol 2005 23 9067 9072 10.1200/JCO.2004.01.0454 16172462

[B47] Suzuki MM Bird A DNA methylation landscapes: provocative insights from epigenomics Nat Rev Genet 2008 9 465 476 10.1038/nrg2341 18463664

[B48] Ushijima T Watanabe N Shimizu K Miyamoto K Sugimura T Kaneda A Decreased fidelity in replicating CpG methylation patterns in cancer cells Cancer Res 2005 65 11 17 15665274

[B49] Feinberg AP Ohlsson R Henikoff S The epigenetic progenitor origin of human cancer Nat Rev Genet 2006 7 21 33 10.1038/nrg1748 16369569

[B50] Laird PW Principles and challenges of genome-wide DNA methylation analysis Nat Rev Genet 2010 11 191 203 10.1038/nrg2732 20125086

[B51] Cotter TG Apoptosis and cancer: the genesis of a research field Nat Rev Cancer 2009 9 501 507 10.1038/nrc2663 19550425

[B52] Taylor RC Cullen SP Martin SJ Apoptosis: controlled demolition at the cellular level Nat Rev Mol Cell Biol 2008 9 231 241 10.1038/nrm2312 18073771

[B53] O'Driscoll M Jeggo PA The role of double-strand break repair - insights from human genetics Nat Rev Genet 2006 7 45 54 10.1038/nrg1746 16369571

[B54] Okada H Mak TW Pathways of apoptotic and non-apoptotic death in tumour cells Nat Rev Cancer 2004 4 592 603 10.1038/nrc1412 15286739

[B55] Li D Da L Tang H Li T Zhao M CpG methylation plays a vital role in determining tissue- and cell-specific expression of the human cell-death-inducing DFF45-like effector A gene through the regulation of Sp1/Sp3 binding Nucleic Acids Res 2008 36 330 341 10.1093/nar/gkm1028 18033804 PMC2248752

[B56] Zelko IN Mueller MR Folz RJ CpG methylation attenuates Sp1 and Sp3 binding to the human extracellular superoxide dismutase promoter and regulates its cell-specific expression Free Radic Biol Med 2010 48 895 904 10.1016/j.freeradbiomed.2010.01.007 20079429 PMC2838251

[B57] Veeck J Esteller M Breast cancer epigenetics: from DNA methylation to microRNAs J Mammary Gland Biol Neoplasia 2010 15 5 17 10.1007/s10911-010-9165-1 20101446 PMC2824126

[B58] Kelley RF Totpal K Lindstrom SH Mathieu M Billeci K Deforge L Pai R Hymowitz SG Ashkenazi A Receptor-selective mutants of apoptosis-inducing ligand 2/tumor necrosis factor-related apoptosis-inducing ligand reveal a greater contribution of death receptor (DR) 5 than DR4 to apoptosis signaling J Biol Chem 2005 280 2205 2212 10.1074/jbc.M410660200 15520016

[B59] Wu J Apoptosis and angiogenesis: two promising tumor markers in breast cancer (review) Anticancer Res 1996 16 2233 2239 8694549

[B60] Fernandez Y Gu B Martinez A Torregrosa A Sierra A Inhibition of apoptosis in human breast cancer cells: role in tumor progression to the metastatic state Int J Cancer 2002 101 317 326 10.1002/ijc.10628 12209955

[B61] Evan GI Vousden KH Proliferation, cell cycle and apoptosis in cancer Nature 2001 411 342 348 10.1038/35077213 11357141

[B62] Kalari S Pfeifer GP Identification of driver and passenger DNA methylation in cancer by epigenomic analysis Adv Genet 2010 70 277 308 full_text 20920752 10.1016/B978-0-12-380866-0.60010-1 PMC2951285

[B63] Ramchandani S Bhattacharya SK Cervoni N Szyf M DNA methylation is a reversible biological signal Proc Natl Acad Sci USA 1999 96 6107 6112 10.1073/pnas.96.11.6107 10339549 PMC26843

