# Supplementary material for: Investigation of DNA damage response and apoptotic gene methylation pattern in sporadic breast tumors using high throughput quantitative DNA methylation analysis technology
Source: Mol Cancer. 2010 Nov 23;9:303. doi: 10.1186/1476-4598-9-303 (PMC3004830; doi:10.1186/1476-4598-9-303)
Supplement: Additional file 1 — Table S1: Listing of Breast tumor samples studied for methylation status of the DDR - apoptotic genes [file 1476-4598-9-303-S1.DOC]

**Table S1**: Listing of Breast tumor samples studied for methylation status of the DDR – apoptotic genes

| **SAMPLE**  **CODE** | **AGE** | **T SIZE** | **NODE** | **STAGE** | **ER** | **PR** | **GRADE** | **SAMPLE**  **CODE** | **AGE** | **T SIZE** | **NODE** | **STAGE** | **ER** | **PR** | **GRADE** |
| --- | --- | --- | --- | --- | --- | --- | --- | --- | --- | --- | --- | --- | --- | --- | --- |
| **P1** | 48 | T4 | N1 | 3B | NEG | NEG | II | **P42** | 49 | T1 | N1 | 2A | NEG | NEG | III |
| **P2** | 68 | T3 | N1 | 3A | ND | ND | ND | **P43** | 55 | T2 | N0 | 2A | NEG | NEG | III |
| **P3** | 25 | T2 | N2 | 3A | ND | ND | ND | **P44** | 53 | T2 | N3 | 3B | NEG | NEG | II |
| **P4** | 25 | T2 | N2 | 3A | POS | NEG | III | **P45** | 53 | T3 | N1 | 3A | NEG | NEG | III |
| **P5** | 62 | T4 | N2 | 3B | POS | POS | II | **P46** | 49 | T1 | N0 | 1 | POS | POS | II |
| **P6** | 42 | T4 | N2 | 4 | POS | POS | II | **P47** | 38 | T2 | N0 | 2A | NEG | NEG | III |
| **P7** | 37 | T2 | N2 | 3A | ND | ND | ND | **P48** | 49 | T3 | N1 | 3A | NEG | NEG | III |
| **P8** | 51 | T4 | N0 | 3A | ND | ND | ND | **P49** | 39 | T2 | N2 | 3A | NEG | NEG | III |
| **P9** | 30 | T4 | N1 | 3A | ND | ND | ND | **P50** | 34 | T2 | N0 | 2A | ND | ND | ND |
| **P10** | 35 | T4 | N1 | 4 | ND | ND | ND | **P51** | 46 | T2 | N0 | 2A | ND | ND | ND |
| **P11** | 68 | T4 | N2 | 3B | NEG | NEG | II | **P52** | 77 | T2 | N2 | 3A | POS | POS | III |
| **P12** | 46 | T2 | N1 | 2B | ND | ND | ND | **P53** | 55 | T2 | N0 | 2A | POS | NEG | III |
| **P13** | 44 | T2 | N2 | 3A | POS | POS | I | **P54** | 49 | T2 | N0 | 2A | ND | ND | ND |
| **P14** | 47 | T4 | N1 | 3A | POS | POS | II | **P55** | 53 | T2 | N1 | 2B | NEG | NEG | III |
| **P15** | 60 | T2 | N0 | 2A | ND | ND | ND | **P56** | 48 | T2 | N1 | 2B | ND | ND | ND |
| **P16** | 44 | T4 | N0 | 3A | ND | ND | ND | **P57** | 45 | T2 | N0 | 2A | ND | ND | ND |
| **P17** | 46 | T4 | N2 | 3B | ND | ND | ND | **P58** | 51 | T3 | N0 | 2B | NEG | NEG | III |
| **P18** | 42 | T2 | N0 | 2A | ND | ND | ND | **P59** | 46 | T2 | N0 | 2A | POS | POS | II |
| **P19** | 56 | T2 | N0 | 2A | ND | ND | ND | **P60** | 45 | T1 | N0 | 1 | NEG | NEG | II |
| **P20** | 37 | T2 | N0 | 2A | ND | ND | ND | **P61** | 45 | T2 | N1 | 2B | ND | ND | ND |
| **P21** | 50 | T3 | N0 | 2B | NEG | NEG | II | **P62** | 72 | T1 | N0 | 1 | POS | NEG | III |
| **P22** | 58 | T2 | N1 | 2A | POS | POS | III | **P63** | 75 | T3 | N3 | 3B | POS | POS | II |
| **P23** | 49 | T2 | N1 | 2B | NEG | NEG | III | **P64** | 57 | T2 | N0 | 2A | ND | ND | ND |
| **P24** | 46 | T2 | N1 | 2B | NEG | NEG | II | **P65** | 39 | T2 | N1 | 2B | ND | ND | ND |
| **P25** | 55 | T2 | N0 | 2A | NEG | NEG | III | **P66** | 45 | T1 | N0 | 1 | NEG | NEG | III |
| **P26** | 49 | T1 | N0 | 1 | POS | POS | II | **P67** | 48 | T2 | N1 | 2B | ND | ND | ND |
| **P27** | 49 | T1 | N1 | 2A | POS | POS | II | **P68** | 52 | T2 | N1 | 2B | POS | NEG | III |
| **P28** | 47 | T2 | N0 | 2A | NEG | NEG | II | **P69** | 53 | T2 | N1 | 2B | NEG | NEG | III |
| **P29** | 45 | T1 | N0 | 1 | POS | POS | II | **P70** | 57 | T2 | N0 | 2A | NEG | NEG | II |
| **P30** | 45 | T1 | N2 | 3A | NEG | NEG | II | **P71** | 48 | T4 | N3 | 3B | POS | POS | III |
| **P31** | 49 | T2 | N0 | 2A | NEG | NEG | III | **P72** | 26 | T3 | N1 | 3A | NEG | NEG | III |
| **P32** | 37 | T2 | N0 | 2A | NEG | NEG | II | **P73** | 55 | T2 | N0 | 2A | POS | POS | II |
| **P33** | 45 | T1 | N1 | 2A | POS | POS | II | **P74** | 35 | T2 | N3 | 3B | ND | ND | ND |
| **P34** | 30 | T2 | N0 | 2A | NEG | NEG | III | **P75** | 27 | T2 | N0 | 2A | POS | POS | III |
| **P35** | 49 | T2 | N0 | 2A | POS | POS | II | **P76** | 45 | T2 | N0 | 2A | POS | POS | II |
| **P36** | 64 | T2 | N0 | 2A | POS | POS | II | **P77** | 55 | T4 | N0 | 3B | NEG | NEG | II |
| **P37** | 49 | T4 | N3 | 3B | NEG | POS | I | **P78** | 36 | T2 | N0 | 2A | POS | POS | II |
| **P38** | 45 | T4 | N0 | 3B | NEG | NEG | II | **P79** | 39 | T2 | N3 | 3B | POS | POS | II |
| **P39** | 49 | T2 | N0 | 2A | NEG | NEG | II | **P80** | 35 | T1 | N1 | 2A | ND | ND | ND |
| **P40** | 66 | T1 | N0 | 1 | POS | POS | II | **P81** | 65 | T4 | N3 | 3B | POS | POS | III |
| **P41** | 45 | T2 | N0 | 2A | NEG | NEG | III |  |  |  |  |  |  |  |  |

ND: Not Determined
