# Supplementary material for: Investigation of DNA damage response and apoptotic gene methylation pattern in sporadic breast tumors using high throughput quantitative DNA methylation analysis technology
Source: Mol Cancer. 2010 Nov 23;9:303. doi: 10.1186/1476-4598-9-303 (PMC3004830; doi:10.1186/1476-4598-9-303)
Supplement: Additional file 2 — Table S2: Tagged methylation primers designed using epidesigner software [file 1476-4598-9-303-S2.DOC]

**Table S2**: Tagged methylation primers designed using epidesigner software

| **SR. NO.** | **AMPLICON** | **PRIMER SEQUENCE** |
| --- | --- | --- |
| **1** | *TRAIL*_10F | 5' aggaagagagAAGTAGGAAAGTTTTTAGAGAGGGGT 3' |
|  | *TRAIL*_T7R | 5' cagtaatacgactcactatagggagaaggctAAAAAAACTTCTTTCAATTTCCCTC 3' |
| **2** | *DR4*_10F | 5' aggaagagagTTTAGATGTATTTTAGTTGGTGGTGG 3' |
|  | *DR4*_T7R | 5' cagtaatacgactcactatagggagaaggctTAAAAACCTCTTAAAAACCAAACCC 3' |
| **3** | *DR5*_10F | 5' aggaagagagATGTGTTTAGGTTGATTTGGGG 3' |
|  | *DR5*_T7R | 5' cagtaatacgactcactatagggagaaggctAAAAACAAAAAAAACAAATACCCCT 3' |
| **4** | *DCR1*_10F | 5' aggaagagagTGTTTGGAAGTGATTGTTGTAAGTG 3' |
|  | *DCR1*_T7R | 5' cagtaatacgactcactatagggagaaggctAATAACCAAAACCAAACATCCCTAC 3' |
| **5** | *DCR2*_10F | 5' aggaagagagGGGTTTAGGAGTTATGGTTTGGTT 3' |
|  | *DCR2*_T7R | 5' cagtaatacgactcactatagggagaaggctTACCTCCTCTAAAACTCACTACCCA 3' |
| **6** | *CASP8*_10F | 5' aggaagagagATTTGTTTTAGAAATAGGGTTGTGG 3' |
|  | *CASP8*_T7R | 5' cagtaatacgactcactatagggagaaggctAAAAAAAACTATACACCTCAAAACATC 3' |
| **7** | *FLIP*_10F | 5' aggaagagagTTTGTTTAGTGATAGTTGAGATAATAAGGA 3' |
|  | *FLIP*_T7R | 5' cagtaatacgactcactatagggagaaggctCCTACAAAACTAAATCAAATTCCAAA 3' |
| **8** | *BCL2*_10F | 5' aggaagagagGGTGGTTTAGAGGAGGGTTTTTT 3' |
|  | *BCL2*_T7R | 5' cagtaatacgactcactatagggagaaggctTCCCTCTTTTCCTAAAAAAAATAACTA 3' |
| **9** | *CYCS*_10F | 5' aggaagagagGTATTGGGATTGGAGTTAATGAGG 3' |
|  | *CYCS*_T7R | 5' cagtaatacgactcactatagggagaaggctAACCACAATCCAAAATCTTCACTC 3' |
| **10** | *ATM*_10F | 5' aggaagagagAGGGAAAATTTTTGGTTTTAAAGGT 3' |
|  | *ATM*_T7R | 5' cagtaatacgactcactatagggagaaggctCCATATCCACCAATAACCAAC 3' |
| **11** | *TP53*_F | 5' aggaagagagTTGATGAGAAGAAAGGATTTAGTTGA 3' |
|  | *TP53*_R | 5' cagtaatacgactcactatagggagaaggctAAAAACTTACCCAATCCAAAAAAAC 3' |
| **12** | *BRCA1*_F | 5' aggaagagagTAATTGGAAGAGTAGAGGTTAGAGGG 3' |
|  | *BRCA1*_R | 5' cagtaatacgactcactatagggagaaggctCCAAAACAAAAAATAAAAACCTCCT 3' |
| **13** | *BRCA2*_10F | 5' aggaagagagTTGGTAGAGATAAAAGGGTAAGAAGT 3' |
|  | *BRCA2*_T7R | 5' cagtaatacgactcactatagggagaaggctAAAAAAAACACACACTCCAACTCC 3' |
| **14** | *CHEK2*_10F | 5' aggaagagagGGTATTGTGGGTAGAAGAATTTGTTT 3' |
|  | *CHEK2*_T7R | 5' cagtaatacgactcactatagggagaaggctAACCAATAAAAAACAACAAATATAACC 3' |
| **15** | *RNF8*_10F | 5' aggaagagagTTGTGTTTTTTGTTTTTGTAGATTTG 3' |
|  | *RNF8*_T7R | 5' cagtaatacgactcactatagggagaaggctTACCTCTATACATCCCCTCAACTTC 3' |
| **16** | *TIP60*_10F | 5' aggaagagagAATGAAGTTTATAGATTTGGGGGAG 3' |
|  | *TIP60*_T7R | 5' cagtaatacgactcactatagggagaaggctAACCCTAAATAAAAACCCCATAAC 3' |
| **17** | *H2AX*_10F | 5' aggaagagagGATTTGGAAAGGAGGGTTGTAGTAG 3' |
|  | *H2AX*_T7R | 5' cagtaatacgactcactatagggagaaggctTATAAACCCCCAAAACACACCTCTA 3' |
