# Supplementary material for: Investigation of DNA damage response and apoptotic gene methylation pattern in sporadic breast tumors using high throughput quantitative DNA methylation analysis technology
Source: Mol Cancer. 2010 Nov 23;9:303. doi: 10.1186/1476-4598-9-303 (PMC3004830; doi:10.1186/1476-4598-9-303)
Supplement: Additional file 3 — Table S3: CpG summary of the 17 genes belonging to the DDR-apoptotic pathway [file 1476-4598-9-303-S3.DOC]

**Table S3**: CpG summary of the 17 genes belonging to the DDR-apoptotic pathway

| **GENE** | **AMPLICON**  **SIZE** | **TOTAL NO. OF**  **CpG SITES IN**  **AMPLICON** | **NO. OF ANALYSED**  **CpG SITES IN**  **AMPLICON** | **PERCENTAGE**  **DETECTION** | **SINGLE**  **SITE**  **CpGs** | **COMPOSITE**  **SITE**  **CpGs** | **COMPOSITE**  **SITES**  **(UNITS)** | **CpG SITES/ UNITS ***  **DETECTED** |
| --- | --- | --- | --- | --- | --- | --- | --- | --- |
| ***TRAIL*** | 479 | 9 | 5 | 55.56 | 3 | 2 | 1 | 4 |
| ***DR4*** | 408 | 24 | 14 | 58.33 | 4 | 10 | 4 | 8 |
| ***DR5*** | 427 | 29 | 10 | 34.48 | 3 | 7 | 3 | 6 |
| ***DCR1*** | 446 | 35 | 22 | 62.86 | 5 | 17 | 6 | 11 |
| ***DCR2*** | 496 | 29 | 12 | 41.38 | 6 | 6 | 3 | 9 |
| ***CASP8*** | 364 | 9 | 7 | 77.78 | 5 | 2 | 1 | 6 |
| ***FLIP*** | 424 | 43 | 28 | 65.12 | 4 | 24 | 8 | 12 |
| ***BCL2*** | 462 | 55 | 10 | 18.18 | 4 | 6 | 2 | 6 |
| ***CYCS*** | 351 | 38 | 19 | 50.00 | 3 | 16 | 7 | 10 |
| ***ATM*** | 473 | 39 | 15 | 38.46 | 9 | 6 | 3 | 12 |
| ***TP53*** | 483 | 17 | 8 | 47.06 | 4 | 4 | 2 | 6 |
| ***BRCA1*** | 370 | 18 | 13 | 72.22 | 8 | 5 | 2 | 10 |
| ***BRCA2*** | 489 | 47 | 13 | 27.66 | 2 | 11 | 5 | 7 |
| ***CHEK2*** | 395 | 27 | 16 | 59.26 | 6 | 10 | 5 | 11 |
| ***RNF8*** | 479 | 28 | 13 | 46.43 | 5 | 8 | 3 | 8 |
| ***TIP60*** | 265 | 22 | 12 | 54.55 | 2 | 10 | 5 | 7 |
| ***H2AX*** | 381 | 23 | 10 | 43.48 | 4 | 6 | 2 | 6 |
| **TOTAL** | 7192 | 492 | 227 | 46.14 | 77 | 150 | 62 | 139 |

* EpiTYPER generates quantitative results for each cleavage product examined. Each cleavage product consists of either one CpG site or a collection of multiple CpG sites. An analyzed unit containing one or multiple CpG sites is termed a “CpG unit”.
