# Supplementary figures and images for: Investigation of DNA damage response and apoptotic gene methylation pattern in sporadic breast tumors using high throughput quantitative DNA methylation analysis technology
Source: Mol Cancer. 2010 Nov 23;9:303. doi: 10.1186/1476-4598-9-303 (PMC3004830; doi:10.1186/1476-4598-9-303)

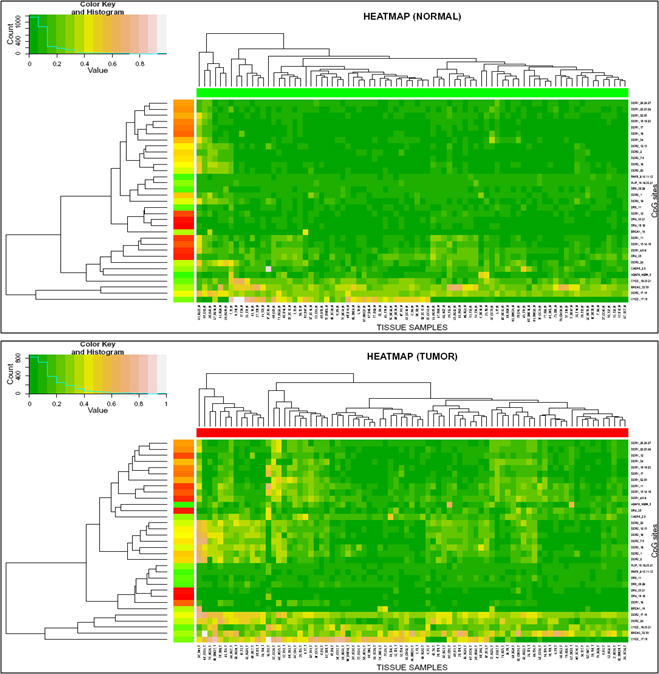

Supplement: Additional file 4 — Figure S1: Heatmap showing differential methylation pattern in breast tumor (red) and normal (green) [file 1476-4598-9-303-S4.JPEG]

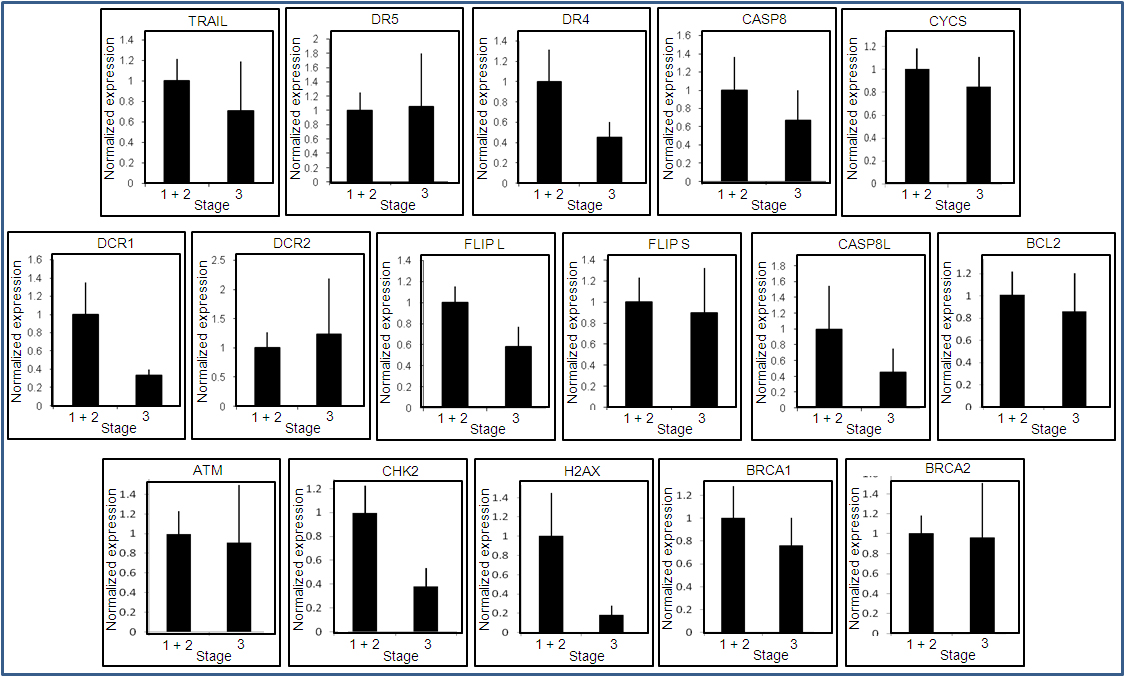

Supplement: Additional file 5 — Figure S2: Categorization of expression pattern of individual death receptor apoptotic pathway and DNA damage response pathway genes stratified with respect to the breast tumor stage [file 1476-4598-9-303-S5.JPEG]
